# Third Window Syndrome: Surgical Management of Cochlea-Facial Nerve Dehiscence

**DOI:** 10.3389/fneur.2019.01281

**Published:** 2019-12-13

**Authors:** P. Ashley Wackym, Carey D. Balaban, Pengfei Zhang, David A. Siker, Jasdeep S. Hundal

**Affiliations:** ^1^Department of Otolaryngology–Head and Neck Surgery, Rutgers Robert Wood Johnson Medical School, New Brunswick, NJ, United States; ^2^Departments of Otolaryngology, Neurobiology, Communication Sciences & Disorders, and Bioengineering, University of Pittsburgh School of Medicine, Pittsburgh, PA, United States; ^3^Department of Neurology, Rutgers Robert Wood Johnson Medical School, New Brunswick, NJ, United States; ^4^Siker Medical Imaging and Intervention, Portland, OR, United States; ^5^Department of Neurosurgery, Rutgers Robert Wood Johnson Medical School, New Brunswick, NJ, United States

**Keywords:** cochlea-facial nerve dehiscence, cognitive dysfunction, dizziness, perilymph fistula, spatial disorientation, superior semicircular canal dehiscence syndrome, traumatic brain injury, vestibular migraine

## Abstract

**Objective:** This communication is the first assessment of outcomes after surgical repair of cochlea-facial nerve dehiscence (CFD) in a series of patients. Pre- and post-operative quantitative measurement of validated survey instruments, symptoms, diagnostic findings and anonymous video descriptions of symptoms in a cohort of 16 patients with CFD and third window syndrome (TWS) symptoms were systematically studied.

**Study design:** Observational analytic case-control study.

**Setting:** Quaternary referral center.

**Patients:** Group 1 had 8 patients (5 children and 3 adults) with CFD and TWS who underwent surgical management using a previously described round window reinforcement technique. Group 2 had 8 patients (2 children and 6 adults) with CFD who did not have surgical intervention.

**Interventions:** The Dizziness Handicap Inventory (DHI) and Headache Impact Test (HIT-6) were administered pre-operatively and post-operatively. In addition, diagnostic findings of comprehensive audiometry, cervical vestibular evoked myogenic potential (cVEMP) thresholds and electrocochleography (ECoG) were studied. Symptoms before and after surgical intervention were compared.

**Main outcome measures:** Pre- vs. post-operative DHI, HIT-6, and audiometric data were compared statistically. The thresholds and amplitudes for cVEMP in symptomatic ears, ears with cochlea-facial nerve dehiscence and ears without CFD were compared statistically.

**Results:** There was a highly significant improvement in DHI and HIT-6 at pre- vs. post-operative (*p* < 0.0001 and *p* < 0.001, respectively). The age range was 12.8–52.9 years at the time of surgery (mean = 24.7 years). There were 6 females and 2 males. All 8 had a history of trauma before the onset of their symptoms. The mean cVEMP threshold was 75 dB nHL (SD 3.8) for the operated ear and 85.7 dB (SD 10.6) for the unoperated ear. In contrast to superior semicircular canal dehiscence, where most ears have abnormal ECoG findings suggestive of endolymphatic hydrops, only 1 of 8 operated CFD ears (1 of 16 ears) had an abnormal ECoG study.

**Conclusions:** Overall there was a marked improvement in DHI, HIT-6 and symptoms post-operatively. Statistically significant reduction in cVEMP thresholds was observed in patients with radiographic evidence of CFD. Surgical management with round window reinforcement in patients with CFD was associated with improved symptoms and outcomes measures.

## Introduction

Ninety years ago, Tullio described the physiologic outcomes of creating a third mobile window in the semicircular canals of pigeons (1). Since that time, many locations of third mobile windows have been described (2–43); however, the sound-induced dizziness and/or nystagmus has been memorialized by the eponym “Tullio phenomenon.” Clinically, the most thoroughly characterized third mobile window is superior semicircular canal dehiscence (SSCD). In 1998, Minor and coworkers first reported the diagnosis of CT positive (CT+) SSCD (10). Minor later reported a conductive hearing loss, which was recognized as a pseudoconductive hearing loss (bone-conduction hyperacusis), as well as a reduced cervical vestibular myogenic potential (cVEMP) threshold in patients with SSCD to 81 ± 9 dB nHL (11). While SSCD is well-recognized; Wackym and colleagues reported the existence of a CT negative (CT–) third window syndrome (TWS) with the same clinical phenotype of SSCD that also exists (12–19). In three published series of such CT– TWS patients (no otic capsule dehiscence visible on imaging) all were treated with round window reinforcement (RWR) (12–14). In these publications, we reported that CT– TWS is associated with a pseudoconductive hearing loss and the abnormally reduced cVEMP threshold, among other objective findings typically found in SSCD patients (12–14). We have proposed the more general term of TWS or otic capsule dehiscence syndrome (OCDS) because the same spectrum of symptoms, signs on physical examination and audiological diagnostic findings are encountered with SSCD, cochlea-facial nerve dehiscence (CFD), cochlea-internal carotid artery dehiscence, cochlea-internal auditory canal dehiscence, lateral semicircular canal-superior semicircular canal ampulla dehiscence, modiolus, “perilymph fistula (PLF),” posterior semicircular canal dehiscence, posterior semicircular canal-jugular bulb dehiscence, SSCD-subarcuate artery dehiscence, SSCD-superior petrosal vein dehiscence, vestibule-middle ear dehiscence, lateral semicircular canal-facial nerve dehiscence, wide vestibular aqueduct in children, post-traumatic hypermobile stapes footplate, otosclerosis with internal auditory canal involvement and in patients with CT– TWS (2–43). A common structural finding in all of these conditions is an otic capsule defect that creates a “third window.”

In 2014, Robert Jyung et al. were the first to identify CFD resulting in TWS; however, neither of their two patients were managed surgically (28). Interest in this clinical entity producing TWS has been mounting, as there have been three recent studies focused on the histologic, CT and cadaveric micro-CT prevalence of CFD (32, 44, 45). The relationship between CFD and facial nerve stimulation in cochlear implant recipients has also been described in a total of 5 patients (46, 47). In the series with 3 patients, no TWS symptoms were presented (46). In the other case report of the other 2 patients they reported that they had no balance problems or autophony; however, no cVEMP data or other TWS symptoms were presented (47). The present report represents the first description of clinical features and outcomes of CFD managed surgically with round window reinforcement (RWR). In addition to comparing the DHI and HIT-6 data, we compared the traditional metrics used in SSCD studies including audiometric data, resolution of symptoms as well as the cVEMP thresholds and amplitudes (8–14, 20, 21, 23, 27, 36, 37, 40, 41). However, because of the tissue placed in the middle ear during the RWR procedure interferes with air-conduction for the cVEMP studies, we did not routinely repeat these studies post-operatively.

## Materials and Methods

### Subjects, Validated Survey Instruments and Surgical Intervention

#### High-Resolution Temporal Bone Computed Tomography (CT) Findings

The OsiriX MD (Pixmeo SARL, Bernex, Switzerland) database built by the neurotologist author (PAW) was used to identify cases of CFD among all of the high-resolution temporal bone CT scans performed in patients with TWS symptoms. Each CT was reviewed by the neurotologist author (PAW) and the neuroradiologist author (DAS) to determine the presence of a CFD and the other known sites of bony dehiscence cataloged in the Introduction; and also to ascertain cases of CT– TWS.

#### Subjects

The procedures followed were in accordance with the ethical standards of the responsible committee on human experimentation and with the Helsinki Declaration. The Rutgers Biomedical Health Sciences Institutional Review Board approved these observational analytic case-control studies (IRB Pro2019000726). The Institutional Review Board granted a consent waiver and also approved the use of age and gender as deidentified data. Inclusion criteria encompassed TWS patients with an otic capsule bony dehiscence limited to CFD. Exclusion criteria included multiple sites of dehiscence, aural atresia, bilateral CFD with only one side operated, <6 months of post-operative follow up, those who did not complete their diagnostic testing and those involved in active litigation. After March 20, 2019, no data were collected from the clinical services provided.

Sixteen subjects with CFD were included in this study. There were two cohorts; Group 1 (CFD with RWR surgery) and Group 2 (CFD without RWR surgery) (Tables 1, 2). The patient demographics and clinical features for each subject are summarized in Tables 1, 2. The 16 patients were not identical in the reported TWS symptoms, but reflected the spectrum of symptoms seen in TWS (Table 3). The laterality of the TWS was determined by the ear that had sound-induced symptoms and heard internal sounds. To further confirm laterality, another useful technique was to ask patients (or parents) to use an ear plug in one ear while exposed to loud sounds or music and to alternate placement of the plug to determine which ear is responsible for sound-induced symptoms. Likewise, encouragement to use an earbud or headphone with sound delivered to individual ears often confirms the ear affected by a third mobile window. Low frequency sounds, particularly with prominent bass components, such as hip-hop music, were particularly useful in inducing symptoms. Clinically, pneumatic otoscopy while a patient wears Frenzel lenses (fistula test/Hennebert sign) was another useful intervention to confirm laterality.

**Table 1:** Patient third window syndrome symptoms, physical findings, and results of diagnostic studies in 16 patients with cochlea-facial nerve dehiscence.

**Group 1: Cochlea-facial nerve dehiscence and third window syndrome patients who underwent round window reinforcement surgery**									
**Patient (age at surgery)**	**Sex**	**Sound-induced**	**Hearing internal sounds**	**128 and 256 Hz tuning forks**	**Pseudoconductive hearing loss**	**Electrocochleography (SP/AP ratio)**	**cVEMP threshold (dB nHL)/amplitude (μV)**	**Surgery performed (length of follow-up)**	**High-resolution TB CT**
1^*^ (12.75)	M	Dizziness and nausea, increased headache	Heartbeat	Negative	None	L 0.36 R 0.32	L 75 dB/437 μV R 95 dB/458 μV	L RWR (52 months)	L CFD
2^*^ (12.92)	M	Increased HA, dizziness, pain	Eyes moving and blinking (R > L)	Positive (back of head)	Left, Right (true conductive hearing loss)	L 0.43 (ELH) R 0.38	L 80 dB/77 μV R 95 dB/301 μV (with true conductive hearing loss)	L RWR R RWR (71 months)	L CT– TWS R CFD
3 (15.17)	F	Dizziness, headache	Eyes blinking, chewing, heel strike	Positive	Bilateral	L 0.38 R 0.46 (ELH)	L 80 dB/121 μV R 75 dB/148 μV	R RWR (10 months)	R CFD L near-CFD
4 (16.5)	F	Increased headache, no dizziness	Voice resonant (left)	Positive (back of head)	Left	L 0.33 R 0.34	L 75 dB/466 μV R 95 dB/358 μV	L RWR (49 months)	L CFD
5^*^ (17.19)	F	Dizziness, migraine; severe sound sensitivity/pain	Eyes blinking, autophony	Positive	Bilateral	L 0.28 R 0.32	L 90 dB/415 μV R 70 dB/619 μV	R RWR (69 months)	R CFD
6^*^ (19.0)	F	Dizziness, nausea, agitated, worsens postural dyscontrol	Voice resonant (L > R), eyes moving and blinking (R), heartbeat (R), chewing (R)	Positive	Bilateral	L 0.39 R 0.37	L 70 dB/194 μV R 70 dB/206 μV	R RWR (11 months)	R CFD
7^*^ (51.42)	F	Dizziness, nausea, HA	Voice resonant, heartbeat	Positive	Left	L 0.36 R 0.35	L 75 dB/277 μV R 75 dB/296 μV	L RWR (12 months)	L CFD
8 (52.92)	F	Dizziness, nausea	Voice resonant	Negative	Bilateral	L 0.14 R 0.37	L 95 dB/3.3 μV R 80 dB/22 μV	R RWR (37 months)	R CFD
**Group 2: Cochlea-facial nerve dehiscence and third window syndrome patients who did not undergo round window reinforcement surgery**									
**Patient (age at presentation)**	**Sex**	**Sound-induced**	**Hearing internal sounds**	**128 and 256 Hz tuning forks**	**Pseudoconductive hearing loss**	**Electrocochleography (SP/AP ratio)**	**cVEMP threshold (dB nHL)/amplitude (μV)**	**Surgery performed**	**High-resolution TB CT**
1 (6.65)	M	Dizziness; pain	Heel strike, face being touched	Positive	Left	L 0.36 R 0.17	L 70 dB/1,093 μV R 70 dB/531 μV	None	Bilateral CFD (R > L)
2 (7.58)	F	Dizziness; pain	Voice resonant	Positive	Left	L 0.26 R 0.30	L 70 dB/430 μV R 80 dB/387 μV	None	L CFD
3 (27.88)	F	Dizziness	Voice resonant	Positive	Left (small)	L 0.30 R 0.29	L 75 dB/180 μV R 80 dB/170 μV	None	L CFD
4 (28.71)	F	Dizziness, increased headache	No	Positive	Bilateral	L 0.39 R 0.23	L 70 dB/554 μV R 90 dB/539 μV	None	Bilateral CFD (L > R)
5 (30.27)	F	Dizziness, confusion, overwhelmed, headache	Eyes blinking, voice resonant, chewing, heartbeat	Positive	Bilateral	L 0.37 R 0.31	L 80 dB/134 μV R NR	None	Bilateral CFD
6 (34.61)	M	Head pain, bitter taste	No	Positive	Left	L 0.37 R 0.39	L 75 dB/400 μV R 80 dB/229 μV	None	L CFD or near-CFD
7 (54.71)	M	Agitated, sense of foreboding	Chewing	Positive	Bilateral	L 0.36 R 0.35	L 80 dB/100 μV R 80 dB/75 μV	None	L CFD
8 (55.67)	F	Dizziness	Chewing	Positive	Left	L NR R NR	L 85 dB/41 μV R 95 dB/20 μV	None	L CFD

* *See video links in references (15–19) [Cohort 1: subject 1 (15), subject 2 (16), subject 5 (17), subject 6 (18), subject 7 (19)]; 128 and 256 Hz = ability to hear or feel the vibration of the tuning fork in the head when applied to knees and elbows; CFD, cochlea-facial nerve dehiscence; CT, computed tomography scan; CT–, CT negative (no dehiscence seen on CT); cVEMP, cervical vestibular evoked myogenic potential; dB nHL, decibel above normal adult hearing level; Dizziness, gravitational receptor asymmetry type of vertigo (e.g., as if on a boat, rocky, wavy, tilting, being pushed, pulled, flipped, or sense of floor falling out from under them); ELH, endolymphatic hydrops (abnormal summating potential/action potential [SP/AP] ratio >0.42 by electrocochleography); F, female; HA, headache; L, left; M, male; TB, temporal bone; R, right. The classification of headache and migraine used in this study followed the International Headache Society's International Classification of Headache Disorders, 3rd edition (ICHD3)*.

**Table 2:** Patient demographics, history, symptoms, and results of diagnostic studies in 16 patients with cochlea-facial nerve dehiscence.

**Group 1: Cochlea-facial nerve dehiscence and third window syndrome patients who underwent round window reinforcement surgery**											
**Patient (age at surgery)**	**Sex**	**Cognitive dysfunction**	**Spatial disorientation**	**Anxiety**	**Nausea**	**Migraine/Migrainous Headache**	**Duration of medical migraine management before surgery**	**Pre-trauma migraine**	**Trauma**	**Surgery performed (length of follow-up)**	**High-resolution TB CT**
1^*^ (12.75)	M	Impaired attention and concentration; dysnomia, agrammatical speech and aprosdia; difficulty reading; Impaired memory	No	No	Yes	Daily migraine HA, infrequent ocular migraine	2.5 months	Rare migraine HA	Football concussion, TWS after vigorous nose blowing during acute sinusitis	L RWR (52 months)	L CFD
2^*^ (12.92)	M	Impaired attention and concentration; difficulty reading; Impaired memory	Rare difficulty with judging distances and sense of detachment	No	No	24/7, light sensitivity	15 months	None	Snowboarding accident, LOC	L RWR R RWR (71 months)	L CT– TWS RCFD
3 (15.17)	F	Impaired attention and concentration; dysnomia, agrammatical speech and aprosdia; Impaired memory	No	No	No	3 days clusters of migraine HA, light sensitivity, occasional ocular migraine	27 months	Childhood migraine HA, infrequent	Mononucleosis/ pneumonia, severe coughing	R RWR (10 months)	R CFD Lnear-CFD
4 (16.5)	F	Impaired attention and concentration; dysnomia, agrammatical speech and aprosdia; difficulty in name finding; difficulty reading; Impaired memory	Mild difficulty judging distances, particularly in cars	No	Once	24/7, light sensitive, vestibular migraine with rotational vertigo, occasional ocular migraine	13 months	None	Concussion, basketball blow to head, sinus infection with vigorous nose blowing	L RWR (49 months)	L CFD
5^*^ (17.19)	F	Impaired attention and concentration; dysnomia, agrammatical speech and aprosdia; slurred speech; difficulty in name finding; Impaired memory	Difficulty judging distances; sense of detachment	No	No	Constant headache and daily migraine HA	21 months		Concussions (3), onset of symptoms after severe vomiting during influenza infection	R RWR (69 months)	R CFD
6^*^ (19.0)	F	Impaired attention and concentration; dysnomia, agrammatical speech and aprosdia; slurred speech; difficulty in name finding; Impaired memory (lost her photographic memory)	Difficulty judging distances; sense of detachment	Sense of impending doom	Yes (constant)	Frequent migraine HA, light sensitivity, ocular migraine (2), vestibular migraine with rotational vertigo	85 months	Migraine HA history began at age 11 years	Concussions (3)	R RWR (11 months)	R CFD
7^*^ (51.42)	F	Impaired attention and concentration; dysnomia, agrammatical speech and aprosdia; slurred speech; difficulty in name finding; slurred speech; difficulty in name finding; Impaired memory	Difficulty in judging distances; sense of detachment; perception of walls breathing	Sense of impending doom	Yes	Daily migraine HA	22 months	None	MVA with airbag deployment	L RWR (12 months)	L CFD
8 (52.92)	F	Impaired attention and concentration; dysnomia, agrammatical speech and aprosdia; Impaired memory	Difficulty in judging distances; sense of detachment; occasional out of body experiences	No	Yes (extreme)	Chronic migraine HA, ocular migraine once monthly, infrequent vestibular migraine with rotational vertigo	16 months	Adult onset migraine HA clusters with menstrual cycle	MVA	R RWR (37 months)	R CFD
**Group 2: Cochlea-facial nerve dehiscence and third window syndrome patients who did not undergo round window reinforcement surgery**											
**Patient (age at presentation)**	**Sex**	**Cognitive dysfunction**	**Spatial disorientation**	**Anxiety**	**Nausea**	**Migraine/Migrainous Headache**	**Duration of medical migraine management before surgery**	**Pre-trauma migraine**	**Trauma**	**Surgery performed**	**High-resolution TB CT**
1 (6.65)	M	Unknown	Unknown	Unknown	Yes	Weekly migraine HA, intermittent vestibular migraine	NA	NA	None	None	Bilateral CFD (R > L)
2 (7.58)	F	Impaired attention and concentration; dysnomia, agrammatical speech and aprosdia; difficulty reading; Impaired memory	Difficulty judging distances	No	No	Weekly migraine HA, vestibular migraine with rotational vertigo 1 time per week	NA	NA	None	None	L CFD
3 (27.88)	F	Mild impaired attention, concentration and memory	No	No	Yes	Daily migraine HA, ocular migraines (2)	NA	Migraine HA 2 times per week, ocular migraine (1)	Taxi trunk lid “slammed” on head	None	L CFD
4 (28.71)	F	Impaired attention and concentration; dysnomia, agrammatical speech and aprosdia; difficulty reading; Impaired memory and forgetful	Difficulty in judging distances; sense of detachment; perception of walls and floor moving	No	Yes	Daily HA with severe migraine HA 2–3 times per week, occasional vestibular migraine with rotational vertigo	NA	None	MVA with mTBI	None	Bilateral CFD (L > R)
5 (30.27)	F	Impaired attention and concentration; dysnomia, agrammatical speech and aprosdia; occasional slurred speech; difficulty reading; Impaired memory	Difficulty in judging distances; sense of detachment; perception of walls moving	No	Yes	Migraine HA 2–3 times per week, occasional perimenstrual vestibular migraine with rotational vertigo, ocular migraine (1)	NA	Onset migraine HA age 5	None	None	Bilateral CFD
6 (34.61)	M	Impaired attention and concentration; dysnomia, agrammatical speech and aprosdia; occasional slurred speech; difficulty in name finding; Impaired memory	Perception of walls swaying; perceives room proportions distorted	No	Yes	Nearly constant migraine HA	NA	“Sinus headaches”	None; onset symptoms after 4 days of Adderall	None	L CFD or near-CFD
7 (54.71)	M	No	No	No	Yes	Vestibular migraine with rotational vertigo, no migraine HA	NA	NA	None	None	L CFD
8 (55.67)	F	Impaired attention and concentration; dysnomia, agrammatical speech and aprosdia; occasional slurred speech; difficulty in name finding; Impaired memory	Difficulty in judging distances	No	Yes	Occasional migraine HA	NA	NA	None	None	L CFD

* *See video links in references (15–19) [Cohort 1: subject 1 (15), subject 2 (16), subject 5 (17), subject 6 (18), subject 7 (19)]; 24/7 = migraine headache present constantly, 24 h per day and 7 days per week while awake; CFD, cochlea-facial nerve dehiscence; CT, computed tomography scan; CT–, CT negative (no dehiscence seen on CT); F, female; HA, headache; L, left; LOC, loss of consciousness; M, male; mTBI, mild traumatic brain injury; MVA, motor vehicle accident; TB, temporal bone; R, right. The classification of headache and migraine used in this study followed the International Headache Society's International Classification of Headache Disorders, 3rd edition (ICHD3)*.

**Table 3:** Spectrum of symptoms, signs or exacerbating factors seen in third window syndrome and diagnostic tools and metrics available to measure these clinically observed phenomenon.

**Category**	**Symptom, sign, or exacerbating factors**	**Diagnostic tools and metrics**
Sound-induced	Dizziness or otolithic dysfunction (see vestibular dysfunction below); nausea; cognitive dysfunction; spatial disorientation; migraine/migrainous headache; pain (especially children); loss of postural control; falls	History; 128 and 256 Hz tuning forks applied to ankles, knees and/or elbows heard or felt in the ear or head; pneumatic otoscopy; cVEMP/oVEMP with reduced threshold with or without increased amplitude, auditory stimuli inducing symptoms; Romberg test while pure tones delivered to individual ear or low frequency tuning fork applied to elbow
Autophony	Resonant voice; chewing; heel strike; pulsatile tinnitus; joints or tendons moving; eyes moving or blinking; comb or brush through hair; face being touched	History
		
Vestibular dysfunction	Gravitational receptor (otolithic) dysfunction type of vertigo (rocky or wavy motion, tilting, pushed, pulled, tilted, flipped, floor falling out from under); mal de débarquement illusions of movement	History; Dizziness Handicap Inventory (DHI); cVEMP/oVEMP; computerized dynamic posturography; Romberg/sharpened Romberg; head tilt; nuchal muscle tightness
Headache	Migraine/migrainous headache; migraine variants (ocular, hemiplegic or vestibular [true rotational vertigo]); coital cephalagia; photophobia; phonophobia; aura; scotomata	History; Headache Impact Test (HIT-6); Migraine Disability Assessment Test (MIDAS)
		
Cognitive dysfunction	General cognitive impairment, such as mental fog, dysmetria of thought, mental fatigue; Impaired attention and concentration, poor multitasking (women > men); Executive dysfunction; Language problems including dysnomia, agrammatical speech, aprosidia, verbal fluency; Memory difficulties; Academic difficulty including reading problems and missing days at school or work; Depression and anxiety	History ***Cognitive Screen:*** MoCA and Schmahmann syndrome scale ***IQ:*** WRIT or WAIS2 ***Attention:*** NAB, Attention Module and/or CPT3 ***Memory:*** CVLT2, WMS4, or WRAML2 ***Executive Functioning:*** WCST, TMT, D-KEFS ***Language:*** NAB, Naming ***Visuospatial:*** Benton JLO ***Mood/personality:*** Clinical interview, PHQ-9, GAD-7, ACES, BDI2, BAI, Personality Assessment Inventory (PAI), or Millon Behavioral Diagnostic
Spatial disorientation	Trouble judging distances; detachment/passive observer when interacting with groups of people; out of body experiences; perceiving the walls or floor moving	History; subjective visual vertical
		
Anxiety	Sense of impending doom	History; GAD-7; BAI
Autonomic dysfunction	Nausea; vomiting; diarrhea; lightheadedness; blood pressure lability; change in temperature regulation; heart rate lability	History; autonomic testing
		
Endolymphatic hydrops	Ear pressure/fullness not relieved by the Valsalva maneuver; barometric pressure sensitivity	History; Electrocochleography, tympanometry
		
Hearing	Pseudoconductive hearing loss (bone-conduction hyperacusis)	Comprehensive audiometric evaluation including tympanometry, stapedial reflex testing, speech perception testing, air-conduction and bone-conduction thresholds; magnitude varies by site of dehiscence

*ACES, Adverse Childhood Experiences Scale; BAI, Beck Anxiety Inventory; BDI2, Beck Depression Inventory, 2nd edition; Benton JLO, Benton Judgment of Line Orientation; CPT3, Continuous Performance Test, 3rd edition; CVLT2, California Verbal Learning Test, 2nd edition; D-KEFS, Delis-Kaplan Executive Function System; DHI, Dizziness Handicap Inventory; GAD-7, Generalized Anxiety Disorder Screener; HIT-6, Headache Impact Test; MoCA, Montreal Cognitive Assessment; NAB, Neuropsychological Assessment Battery; PAI, Personality Assessment Inventory; PHQ-9, Patient Health Questionnaire; TMT, Trail Making Test; WAIS2, Wechsler Adult Intelligence Scale, 2nd edition; WCST, Wisconsin Card Sorting Test; WMS4, Wechsler Memory Scale, 4th edition; WRAML2, Wide Range Assessment of Memory and Learning, 2nd edition; WRIT, Wide Range Intelligence Test*.

For those with CFD who had RWR (Group 1), there were 5 children and 3 adults, and a F:M ratio of 6:2. There were 3 patients with a left CFD, 5 with a right CFD, 1 with an asymptomatic left near-CFD and in 1 subject, a left-sided CT– TWS was also present. The mean age at the time of RWR surgery was 24.3 years (range 12.8–52.9 years). The mean duration of follow-up after RWR surgery was 55 months (4 years and 5 months) with a range of 10–71 months.

For those with CFD and who did not have RWR (Group 2), there were 5 children and 3 adults, and a F:M ratio of 5:3. There were 3 bilateral CFD and 4 left CFD. The mean age at the time of initial presentation was 30.8 years (range 6.7–55.7 years).

#### Dizziness Handicap Inventory

As a routine part of their clinical care, all 16 subjects completed the DHI. The DHI is a 25-item self-assessment inventory designed to evaluate the self-perceived handicapping effects imposed by dizziness/vestibular dysfunction. There is a maximum score of 100 and a minimum score of 0. The higher the score, the greater the perceived handicap due to dizziness. For the subjects who underwent RWR, the DHI was also repeated 3–4 months after their final surgical procedure. For the subjects who did not elect surgical intervention, the DHI was performed at their initial evaluation and repeated at their routine follow-up appointment 3–6 months later. The DHI questionnaire responses were entered into each medical record by a nurse not involved with the clinical research and scored automatically via the electronic medical record DHI programming using the scoring system validated by Jacobson and Newman for this instrument (“Yes” = 4 points; “Sometimes” = 2 points; “No” = 0 points) (48). A score of 0–30 indicates mild impairment, a score of 31–60 indicates moderate impairment and a score of 61–100 indicates severe impairment (49). The pre- and post-treatment scores were then totaled, both for the combined total and for each domain score (physical, functional, emotional), difference scores were calculated, and all total scores were entered into an Excel database for analysis. All data were examined with standard descriptive statistics (mean, SD, range). When comparisons between the pre- and post-treatment scores were made in the RWR surgery group, as well as with the initial scores and follow-up scores in the no surgery group, the data were analyzed using repeated-measures analysis of variance and least significant differences tests for paired comparisons, establishing 0.05 as the criterion level of significance.

Statistical comparisons to answer the question, “do specific items change between the two DHI test applications in the CFD cohort who did not choose to have surgery group?,” tested the hypothesis that there are significant differences in individual symptom report scores in that group in the early vs. later tests. This hypothesis was tested by paired *t*-tests (Bonferroni-corrected *p*-value for multiple tests, *p*_corrected_ = 0.05/31 tests) between the two test scores.

Statistical comparisons were made to determine if specific DHI scores by individual question changed between the pre- and post-treatment scores in the RWR surgery group. This hypothesis was tested by paired *t*-tests (Bonferroni-corrected *p*-value for multiple tests) between the two test scores.

#### Headache Impact Test

As a routine part of their clinical care, all 16 subjects completed the HIT-6. The HIT-6 is a six-item self-assessment questionnaire used to measure the impact headaches have on a patient's ability to function on the job, at school, at home and in social situations. For the subjects who underwent RWR, the DHI was also repeated 3–4 months after their final surgical procedure (Tables 1, 2). For the subjects who did not elect surgical intervention, the HIT-6 was performed at their initial evaluation and repeated at their routine follow-up appointment 3–6 months later. The HIT-6 questionnaire responses were entered into each medical record by a nurse not involved with the clinical research and scored automatically via the electronic medical record HIT-6 programming using the scoring system validated for this instrument (“Never” = 6 points; “Rarely” = 9 points; “Sometimes” = 10 points; “Very often” = 11 points; “Always” = 13 points) (50, 51). The final HIT-6 score was obtained from simple summation of the six items and ranges between 36 and 78, with larger scores reflecting greater impact. Headache impact severity level was categorized using score ranges based on the HIT-6 interpretation guide (50, 51). The four headache impact severity categories are little or no impact [49 or less, (Class I)], some impact [50–55, (Class II)], substantial impact [56–59, (Class III)], and severe impact [60–78, (Class IV)]. The pre- and post-treatment scores were examined with standard descriptive statistics (mean, SD, range). When comparisons between the pre- and post-treatment scores were made, the data were analyzed using repeated-measures analysis of variance and least significant differences tests for paired comparisons, establishing 0.05 as the criterion level of significance. The classification of headache and migraine used in this study followed the International Headache Society's International Classification of Headache Disorders, 3rd edition (ICHD3) (52).

Statistical comparison to answer the question, “do specific items change between the two HIT-6 test applications in the CFD cohort who did not choose to have surgery group?” tested the hypothesis that there are significant differences in individual symptom report scores in that group in the early vs. later tests. This hypothesis was tested by paired *t*-tests (Bonferroni-corrected *p*-values for multiple tests) between the two test scores.

Statistical comparisons were made to determine if specific HIT-6 scores by individual question changed between the pre- and post-treatment scores in the RWR surgery group (Group 1) and between the initial evaluation and at their routine follow-up appointment 3–6 months later for Group 2 (the subjects who did not elect surgical intervention). This hypothesis was tested by paired *t*-tests (Bonferroni-corrected *p*-values for multiple tests) between the two test scores.

#### Round Window Reinforcement With the Perichondrial and Cartilage Graft Technique

For CFD patients who had RWR and the 1 patient with right-sided CT– TWS (Group 1, Tables 1, 2), the perichondrial and cartilage graft technique described previously for RWR was ultimately performed in all 8 subjects (12–14). For details, see Supplementary Material.

### Hearing and Balance Testing

#### Comprehensive Audiometric Testing

Pure-tone audiometry was performed over the frequency ranges of 250–8,000 Hz for air conduction and 250–3,000 Hz for bone conduction. Testing was performed in a sound-proof booth. Appropriate masking was used for bone conduction and, when needed, for air conduction. Tympanometry was also performed, and acoustic reflexes were tested for ipsilateral and contralateral presentation of tones. After noting the presence of a pseudoconductive hearing loss, a 4-frequency (500, 1,000, 2,000, and 4,000 Hz) air-bone gap was calculated before and after RWR and presented using the standardized format for reporting hearing outcome in clinical trials (53).

#### Tuning Fork Testing

As a screening tool for patients with TWS symptoms, low-frequency tuning forks were applied to the knees and elbows, and they were asked if they could hear or feel the vibration in their head; 128 and 256 Hz tuning forks were used (54). In addition, for most patients, they stood with feet together, and when possible with eyes closed, while a 256 Hz tuning fork was applied to the elbow on the side in which they most loudly heard or felt the vibration. This typically resulted in a sense of tilting and increased sway (18).

#### Cervical Vestibular Evoked Myogenic Potentials (cVEMP)

A commercial auditory evoked potential software system (ICS Chartr EP 200, Otometrics, Natus Medical Inc., Schaumburg, IL) was used for acoustic cVEMP testing. Sound stimuli were delivered monaurally via an intra-auricular transducer with foam earphones (E-A-R Link Insert Earphones; E-A-R Auditory Systems, Indianapolis) as described previously (55). Peak-to-peak amplitude was calculated with the Otometrics software after peaks were labeled and the amplitude difference between the two peaks was measured. The threshold was defined as the lowest dB nHL at which a p13 and n23 response could be recorded.

#### Electrocochleography (ECoG)

Pre-operative ECoG was performed with gold foil tiptrodes (Etymotic Research; Elk Grove Village, Ill.), which were placed adjacent to the tympanic membrane in the external auditory canal and stabilized at the foam tip of the insert audio transducer. Unfiltered clicks of 100 μs duration were presented at an intensity of 85 dB nHL. Two replications of averaged responses elicited by 1,500 clicks presented at a rate of 11.7/s were obtained. Responses were band-pass filtered (20–1,500 Hz) and averaged, and the summating potential to action potential (SP/AP) ratio was calculated. An SP/AP ratio of >0.42 was defined as abnormal for purposes of this study, based on commonly used standards for clinical testing (56).

### Statistical Analyses

Statistical analyses were conducted with IBM SPSS Statistics for Windows, Version 24.0 (Armonk, NY: IBM Corp.), with Python and R extensions. The individual tests performed, results and associated *p*-values are presented in the text.

## Results

### Subjects, Validated Survey Instruments, and Surgical Intervention

Tables 1, 2 summarize the pre-operative history, symptoms, physical findings and results of diagnostic studies in the 8 patients with CFD who underwent RWR surgery (Group 1). It should be noted that subjects 1, 2, 4, 5, and 8 were previously unrecognized CFD patients who had RWR for what was thought to be a CT– TWS. Tables 1, 2 also summarize the pre-operative history, symptoms, physical findings and results of diagnostic studies in the 8 patients with CFD who did not undergo RWR surgery (Group 2). By 6 months post-operatively, no patients had persistent sound-induced dizziness (Tullio phenomenon) or autophony.

#### High-Resolution Temporal Bone Computed Tomography Findings

The OsiriX MD database built by the neurotologist author (PAW) included 860 studies. Of these, 401 were high-resolution temporal bone CT scans of both temporal bones that were performed to evaluate patients with TWS symptoms. Of the 802 individual temporal bones reviewed, the distribution of otic capsule defects/dehiscence visualized and associated with third window syndrome symptoms in 502 bones were (Table 4): SSCD [175]; near-SSCD [121]; CT– TWS [97]; CFD [52]; SSCD and CFD [30]; cochlea-internal auditory canal [5]; CFD and cochlea-internal auditory canal [4]; lateral semicircular canal dehiscence [3]; wide vestibular aqueduct [3]; CFD and wide vestibular aqueduct [2]; posterior semicircular canal [2]; SSCD-superior petrosal sinus [2]; SSCD and posterior semicircular canal and wide vestibular aqueduct [1]; SSCD-subarcuate artery [1]; SSCD and cochlea-internal auditory canal [1]; SSCD and posterior semicircular canal [1]; posterior semicircular canal-jugular bulb [1]; and the modiolus [1]. The SSCD and CT– TWS temporal bones were counted independent of each other; however, there were 22 that had SSCD plugging that later developed CT– TWS.

**Table 4:** Prevalence of radiographic sites of dehiscence in 502 temporal bones associated with third window syndrome in 401 patients (802 temporal bones).

**Location(s)/Site(s)**	**Prevalence (%)**
Superior semicircular canal dehiscence	175/502 (34.9%)
Near-superior semicircular canal dehiscence	121/502 (24.1%)
CT– third window syndrome	97/502 (19.3%)
Cochlea-facial nerve dehiscence	52/502 (10.4%)
Superior semicircular canal dehiscence + Cochlea-facial nerve dehiscence	30/502 (5.98%)
Cochlea-internal auditory canal dehiscence	5/502 (1.0%)
Cochlea-internal auditory canal dehiscence + Cochlea-facial nerve dehiscence	4/502 (0.8%)
Lateral semicircular canal dehiscence	3/502 (0.6%)
Wide vestibular aqueduct	3/502 (0.6%)
Wide vestibular aqueduct + Cochlea-facial nerve dehiscence	2/502 (0.4%)
Posterior semicircular canal dehiscence	2/502 (0.4%)
Superior semicircular canal-Superior petrosal sinus dehiscence	2/502 (0.4%)
Superior semicircular canal dehiscence + Posterior semicircular canal dehiscence + Wide vestibular aqueduct	1/502 (0.2%)
Superior semicircular canal-Subarcuate artery dehiscence	1/502 (0.2%)
Superior semicircular canal dehiscence + Cochlea-internal auditory canal dehiscence	1/502 (0.2%)
Superior semicircular canal dehiscence + Posterior semicircular canal dehiscence	1/502 (0.2%)
Posterior semicircular canal-Jugular bulb dehiscence	1/502 (0.2%)
Modiolus	1/502 (0.2%)

*CT–, High-Resolution Temporal Bone Computed Tomography Scan Negative for a Visible Site of Dehiscence*.

**Figure 1:**
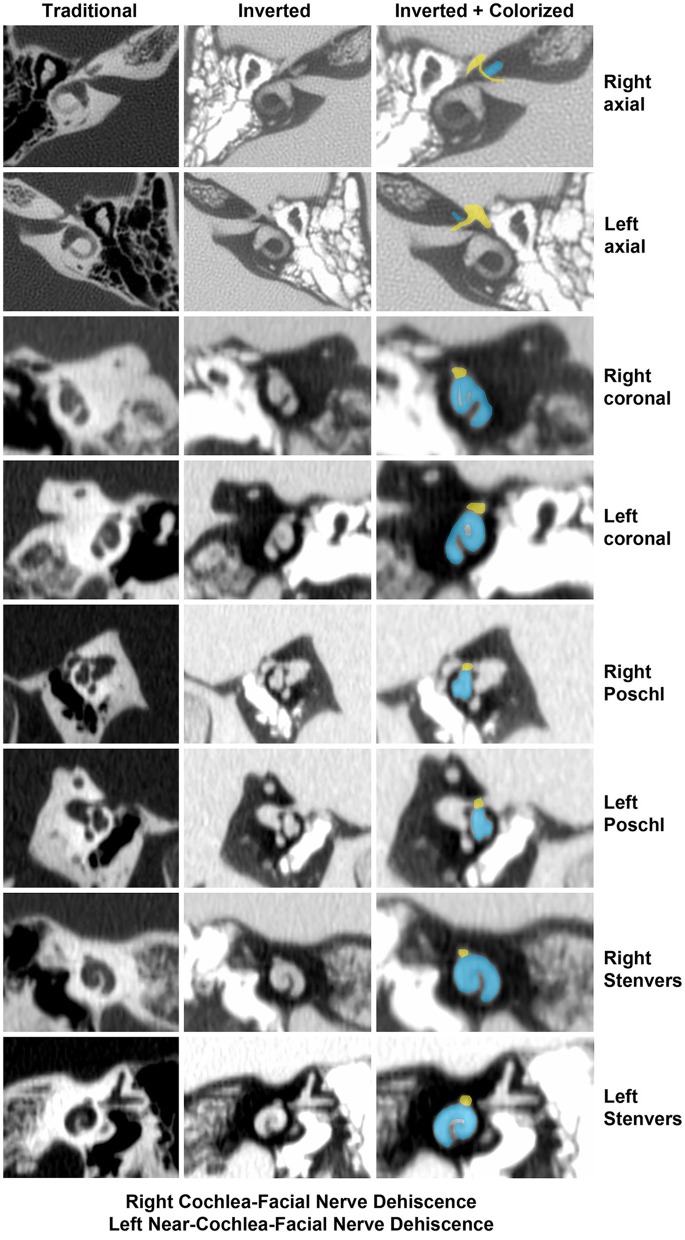
High-resolution temporal bone CT without contrast (Table 1, Group 1 Patient 3). Traditional CT images are shown on the far left column. Cochlea (blue) and facial nerve (yellow) have been colorized and superimposed over inverted images in the axial, coronal, Pöschl and Stenvers planes for both the left and right ears. Note that a cochlea-facial nerve dehiscence (CFD) is seen on the left and a near-CFD is seen on the right. The patient has no left-sided third window syndrome symptoms, with resolution of her third window syndrome symptoms after round window reinforcement on the right. Copyright ©P.A. Wackym, used with permission.

**Figure 2:**
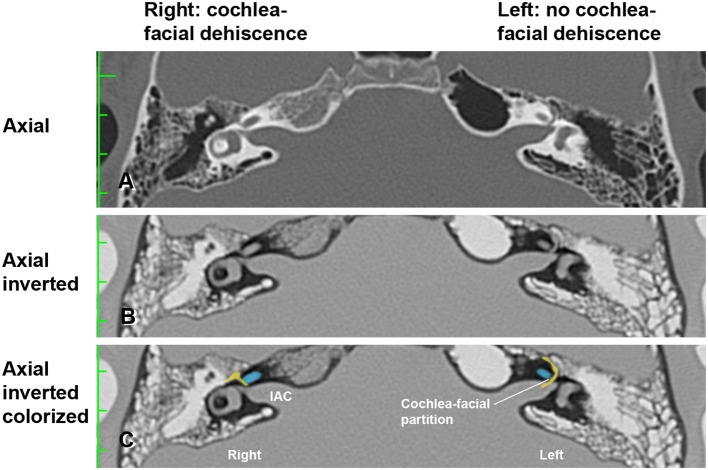
High-resolution temporal bone CT without contrast (Table 1, Group 1 Patient 2). **(A)** Traditional axial CT images are shown. **(B)** Inverted axial CT images. **(C)** Cochlea (blue) and facial nerve (yellow) have been colorized and superimposed over inverted images in the axial plane for both the left and right ears. Note that a cochlea-facial nerve dehiscence (CFD) is seen on the right and a cochlea-facial partition between the cochlea and the facial nerve is seen on the left. The patient had left-sided third window syndrome (TWS) symptoms due to a CT negative TWS, with resolution of his TWS symptoms after round window reinforcement on the right and left. IAC, internal auditory canal. Copyright © P.A. Wackym, used with permission.

Two illustrative cases are shown in Figures 1, 2. In Figure 1, the images showed a right CFD and a left near-CFD or CFD (Tables 1, 2, Group 1 Patient 3). There are several important points regarding this case that should be emphasized. First, only the right ear had TWS symptoms (sound-induced dizziness and headache; autophony [hearing her eyes blinking, chewing sounding loud in her right ear and hearing her heel strike while walking]), but the left side showed radiographic evidence of a possible CFD and a reduced cVEMP threshold of 80 dB nHL. This underscores the need for clinical judgment and decision-making that integrates clinical symptoms, radiographic features and objective test data before surgical intervention should be pursued. She also was the only patient with electrocochleographic evidence of endolymphatic hydrops in the CFD with RWR surgery group. One notes that it is essential, when possible, to visualize the CFD in the axial, coronal, Pöschl and Stenvers views to minimize the possibility that the appearance of the CFD is a partial volume averaging artifact of the image reconstruction algorithms.

In Figure 2, the axial CT images of a male patient with bilateral TWS is shown (Tables 1, 2, Group 1 Patient 2). He had a right CFD and a left CT– TWS that became symptomatic after a snowboarding accident. Bilateral RWR was performed. The images illustrate the CFD on the right and the normally present bony cochlea-facial partition on the left.

#### Subjects and Surgical Intervention

There were 16 subjects who met the inclusion and exclusion criteria and form the two cohorts included in this study. As shown in Tables 1, 2, there were 8 ears that had RWR procedures performed for 3 left CFD and 5 right CFD. One ear (Tables 1, 2, Group 1 Patient 2) had RWR for a CT– TWS. For the cohort with CFD who were not managed surgically (*n* = 8) (Tables 1, 2), 3 had bilateral CFD while the remaining 5 had left CFD.

#### Dizziness Handicap Inventory

**Figure 3:**
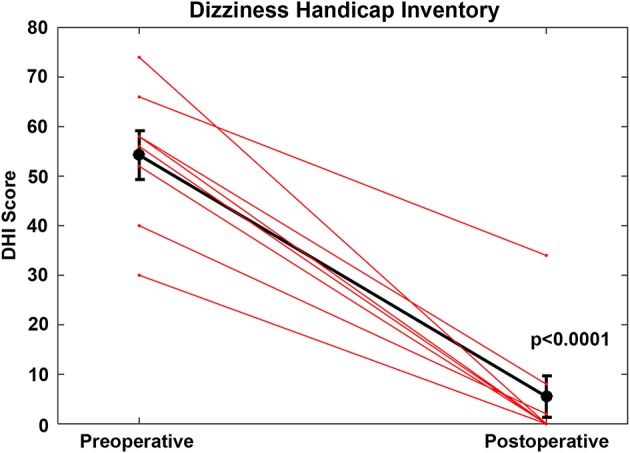
For the cochlea-facial nerve dehiscence cohort who had round window reinforcement procedures performed, the pre-operative mean DHI score was 54.25 (SE 4.9, range 30–74). The post-operative mean DHI score was 5.5 (SE 4.2, range 0–34). This improvement was highly statistically significant (paired *t*-test, *p* < 0.0001). These data are plotted as a single black line. Individual patients are plotted as separate lines (red). Copyright © P.A. Wackym, used with permission.

For the CFD cohort who had RWR procedures performed (Group 1, Tables 1, 2), the pre-operative mean DHI score was 54.3 (SE 4.9, range 30–74). Using the clinical categorical descriptors of the DHI (46, 47), one was at the upper border of mild impairment (score of 30), five subjects had moderate impairment (scores of 40–58) and two subjects had severe impairment (scores >60). The post-operative mean DHI score was 5.5 (SE 4.2, range 0–34), with one subject decreasing from severe to moderate (66 pre-operatively to 34 post-operatively) and the remaining seven showing reductions to the mild range (scores of 0–8). This improvement was highly significant statistically (paired *t*-test, *p* < 0.0001) (Figure 3).

Statistical comparisons were made to determine if specific DHI item scores changed between the pre- and post-treatment scores in the RWR surgery group. This hypothesis was tested by paired *t*-tests (Bonferroni-corrected *p*-values for multiple comparisons) between the two test scores. The following items showed significant improvement after surgery:

P4. *Does walking down the aisle of a supermarket increase your problems?* (*p* = 0.001)
F6. *Does your problem significantly restrict your participation in social activities, such as going out to dinner, going to the movies, dancing, or going to parties?* (*p* = 0.000)
F7. *Because of your problem, do you have difficulty reading?* (*p* = 001)
P8. *Does performing more ambitious activities, such as sports, dancing, household chores (sweeping or putting dishes away) increase your problems?* (*p* = 0.000)
F14. *Because of your problem, is it difficult for you to do strenuous homework or yard work?* (*p* = 0.000)
E18. *Because of your problem, is it difficult for you to concentrate?* (*p* = 0.000)
E21. *Because of your problem, do you feel handicapped?* (*p* = 0.001)
F24. *Does your problem interfere with your job or household responsibilities?* (*p* = 0.000)

As further evidence of the effectiveness of the surgery, the post-operative scores on DHI items P8, F14 and E21 were lower in the operated CFD cohort (*t*-tests, *p* < 0.05) than the second (repeat) test scores for the CFD cohort who did not choose to have surgery.

For the CFD cohort who did not choose to have surgery (Group 2, Tables 1, 2), the initial mean DHI score was 36.5 (SE 10.6, range 0–100). Four subjects showed mild impairment (scores: 0–26), two subjects reported moderate impairment (scores: 34–36) and two subjects reported severe impairment (scores: 62 and 100). At the second administration, the mean DHI score was 42.5 (SE 11.1, range 12–100). There was no statistically significant difference between the initial and second administration of the DHI in the CFD patients who did not elect to undergo surgery (*p* > 0.05). There were no significant changes on any DHI item between the first and second tests.

Statistical comparison of Group 1 (CFD with RWR) to Group 2 (CFD without RWR) (Tables 1, 2) revealed that the DHI scores at initial presentation were no different between the groups (*p* > 0.05). Further, to determine if there were any significant differences between symptom item endorsements in patients that may be related to election of surgery, we tested the hypothesis that there are significant differences in the first symptom report scores (patterns) between the two patient groups. There were three questions that had a statistically significant difference between the groups: P8 [*F*_(1,14)_ = 5.478, *p* < 0.04], F14 [*F*_(1,14)_ = 6.725, *p* < 0.03], and E21 [*F*_(1,14)_ = 5.6, *p* < 0.04]. For item P8, in the cohort who elected not to have surgery, 2 of 8 had a score of 0, while none electing RWR surgery had a score of 0. For item F14, in the cohort who elected not to have surgery, 3 of 8 had a score of 0, while none electing RWR surgery had a score of 0. For item E21, in the cohort who elected not to have surgery, 6 of 8 had a score of 0; 1 of 8 electing RWR surgery had a score of 0. By binary logistic regression (Wald criterion), P4 and F14 were sufficient to classify 7 of 8 of each group correctly, with F14 alone producing a correct classification of 6 of 8 from each group.

#### Headache Impact Test

**Figure 4:**
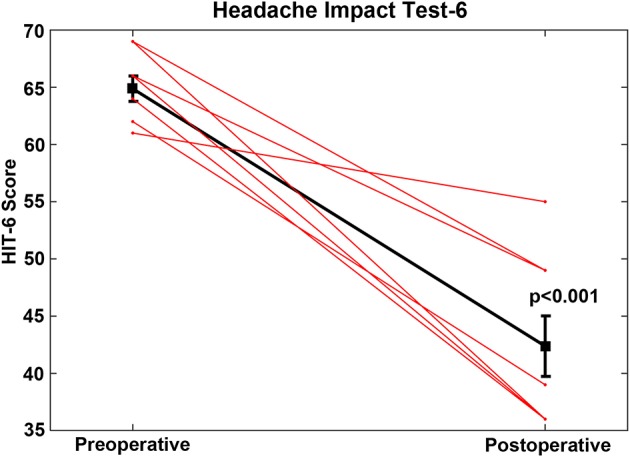
For the cochlea-facial nerve dehiscence cohort who had round window reinforcement procedures performed, the pre-operative mean HIT-6 score was 64.9 (SE 1.1, range 52–69). The post-operative mean HIT-6 score was 42.4 (SE 2.7, range 36–55). This improvement was highly statistically significant (paired *t*-test, *p* < 0.001). These data are plotted as a single black line. Individual patients are plotted as separate lines (red). Copyright © P.A. Wackym, used with permission.

For the CFD cohort who had RWR procedures performed (Group 1, Tables 1, 2), the pre-operative mean HIT-6 score was 64.9 (SE 1.1, range 52–69) and all scores were in the severe impact range (Class IV). The post-operative mean HIT-6 score was 42.4 (SE 2.7, range 36–55); seven subjects shifted into the little or no impact range (<50) (Class I or II) and one subject had a score categorized as Class III. This improvement was highly statistically significant statistically (paired *t*-test, *p* < 0.001) (Figure 4). For all of the CFD patients who elected to undergo RWR, they were treated medically as migraine/vestibular migraine patients without resolution of their symptoms before surgical intervention. The duration of medical management ranged from 2.5 to 85 months (mean = 25.2 months).

Statistical comparisons were made to determine if specific HIT-6 scores for individual questions changed between the pre- and post-treatment scores in the RWR surgery group. This hypothesis was tested by paired *t*-tests (Bonferroni-corrected *p*-values) between the two test scores. The following items showed significant improvement after surgery:

HIT-6 Question 2: *How often do headaches limit your ability to do usual daily activities including household work, work, school, or social activities?* (*p* = 0.000)
HIT-6 Question 3: *When you have a headache, how often do you wish you could lie down?* (*p* = 0.000)
HIT-6 Question 4: *In the past 4 weeks, how often have you felt too tired to do work or daily activities because of your headaches?* (*p* = 0.000)
HIT-6 Question 5: *In the past 4 weeks, how often have you felt fed up or irritated because of your headaches?* (*p* = 0.001)

For the CFD cohort (Group 2) who did not choose to have surgery (Tables 1, 2), the mean HIT-6 score was initially 61.5 (SE 2.9, range 46–76) and at second administration the mean HIT-6 score was 63.1 (SE 2.9, range 49–76). There was no statistically significant difference between the initial and second administration of the HIT-6 in the CFD patients who did not elect to undergo surgery (*p* > 0.05). Four subjects had scores in the severe impact range, with 1 subject in the significant impact range, 2 subjects in the some impact range, and the remaining subject showing “no or little” impact.

Statistical comparisons to answer the question, “do specific items change between the two HIT-6 test applications in the CFD cohort who did not choose to have surgery group?,” tested the hypothesis that there are significant differences in individual symptom report scores in that group in the early vs. later tests. There were no significant changes on any HIT-6 item.

Statistical comparison of Group 1 (CFD with RWR) to Group 2 (CFD without RWR) (Tables 1, 2) revealed that the initial HIT-6 scores were no different between the groups (*p* > 0.05).

### Hearing and Balance Testing

#### Comprehensive Audiometric Testing

**Figure 5:**
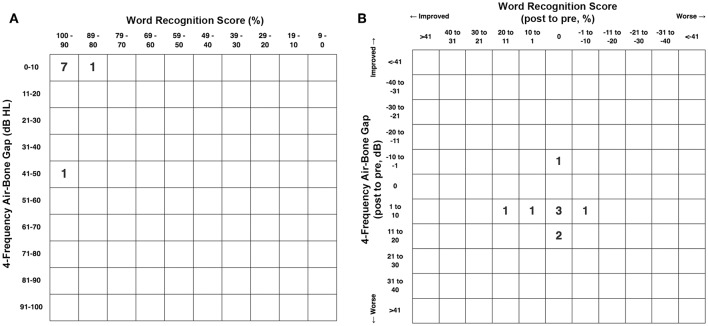
**(A)** Pretreatment scattergram of audiometric data for the 8 patients (9 ears) who underwent round window reinforcement (RWR) for management of cochlea-facial nerve dehiscence (CFD) (*n* = 8, Group 1) and CT negative (CT–) third window syndrome (TWS) (*n* = 1). Seven of the 9 ears had a 4-frequency air-bone gap/pseudoconductive hearing loss between 2.5 and 8.75 dB (mean 5.63 dB). One subject (Table 1, Group 1 Patient 2) had a true conductive hearing loss with a pretreatment 4-frequency air-bone gap of 42.5 dB in his right ear; which had the CFD (see Figure 2). His other ear with the CT– TWS had a pretreatment air-bone gap of 6.25 dB and his pretreatment speech discrimination score was 88% on the left that improved to 100% post-treatment. Copyright © P.A. Wackym, used with permission. **(B)** Post-treatment scattergram of audiometric data for 9 patients who underwent round window reinforcement (RWR) for management of cochlea-facial nerve dehiscence (CFD) (*n* = 8) and CT negative (CT–) third window syndrome (TWS) (*n* = 1) in 8 subjects. Six ears had no change in word recognition score (WRS), including the 1 ear with a true conductive hearing loss and CFD (Patient 2). This same subject (Patient 2) had a CT– TWS (Figure 2) on the left and had a pretreatment speech discrimination score of 88% that improved to 100% post-treatment. Excluding the ear of Patient 2 with the true conductive hearing loss, the pseudoconductive hearing loss with the added conductive hearing loss as a result of the RWR procedure had a mean 4-frequency air-bone gap of 10.94 dB (range 5–23.75 dB). As shown in the scatterplot, 8 ears had worsening of the air-bone gap; while only Patient 5 had an improvement from 7.5 to 5 dB for the 4-frequency air-bone gap. This likely represents the test-retest variability. There was no statistically significant difference in the 4-frequency air-bone gap pretreatment compared to post-treatment (*p* = 0.091). Copyright ^©^P.A. Wackym, used with permission.

Figure 5A shows the pretreatment scattergram of the audiometric data for the 8 patients (9 ears) who underwent RWR for management of their CFD (*n* = 8) and CT– TWS (*n* = 1). Seven of the 9 ears had a 4-frequency air-bone gap/pseudoconductive hearing loss between 2.5 and 8.75 dB (mean 5.63 dB). One subject (Patient 2) had a true conductive hearing loss with a pretreatment 4-frequency air-bone gap of 42.5 dB in his right ear; which had the CFD (see Figure 2). His other ear with the CT– TWS had a pretreatment air-bone gap of 6.25 dB and his pretreatment speech discrimination score was 88% on the left that improved to 100% post-treatment. Figure 5B shows the post-treatment scattergram of the audiometric data who underwent RWR for management of CFD (*n* = 8) and CT– TWS (*n* = 1) in 8 subjects. Six ears had no change in word recognition score (WRS), including the 1 ear with a true conductive hearing loss and CFD (Patient 2). This same subject (Patient 2) had a CT– TWS (Figure 2) on the left and had a pretreatment speech discrimination score of 88% that improved to 100% post-treatment. Excluding the ear of Patient 2 with the conductive hearing loss, the pseudoconductive hearing loss with the added conductive hearing loss as a result of the RWR procedure had a mean 4-frequency air-bone gap of 10.94 dB (range 5–23.75 dB). As shown in the scatter-plot, 8 ears had modest worsening of the air-bone gap; while only Patient 5 had an improvement from 7.5 to 5 dB for the 4-frequency air-bone gap. There was no statistically significant difference in the 4-frequency air-bone gap pretreatment compared to post-treatment (paired *t*-test, *p* = 0.091).

**Figure 6:**
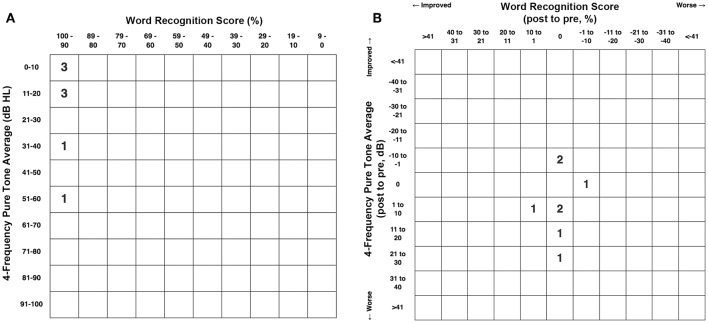
**(A)** Pretreatment scattergram of the 4-frequency air-conduction pure tone average audiometric data for the 8 patients who underwent RWR for management of their CFD (*n* = 8, Group 1). One subject (Table 1, Group 1 Patient 2) had a true conductive hearing loss with a pretreatment 4-frequency air-conduction pure tone average of 56.25 dB in his right ear; which had the CFD (see Figure 2). **(B)** Post-treatment scattergram of the audiometric data for 8 patients who underwent RWR for management of CFD. Six ears had no change in word recognition score (WRS), including the 1 ear with a true conductive hearing loss and CFD (Patient 2). One had an improvement of speech discrimination ability from 96 to 100%, while another had a decrease in speech discrimination from 96 to 92%. Including the ear of Patient 2 with the conductive hearing loss and CFD, the mean pre-operative air-conduction 4-frequency pure tone average was 19.7 dB (range 5–56.25 dB), while the mean post-operative air-conduction 4-frequency pure tone average was 22.8 dB (range 5–51.25 dB). As shown in the scatterplot **(B)**, 5 ears had worsening of the 4-frequency air-conduction pure tone average; while 3 stayed the same or improved post-operatively. There was no statistically significant difference in the 4-frequency air-conduction pure tone average pretreatment compared to post-treatment (paired *t*-test, *p* = 0.472). Six ears had no change in WRS, including the 1 ear with a true conductive hearing loss and CFD (Patient 2). One patient had an improved WRS (96–100%) and one patient had a worsened WRS (96–92%). There was no statistically significant difference in the WRS pretreatment compared to post-treatment (paired *t*-test, *p* = 0.402). Copyright © P.A. Wackym, used with permission.

Figure 6A shows the pretreatment scattergram of the 4-frequency air-conduction pure tone average audiometric data for the 8 patients who underwent RWR for management of their CFD (*n* = 8). One subject (Group 1 Patient 2) had a true conductive hearing loss with a pretreatment 4-frequency air-conduction pure tone average of 56.25 dB in his right ear; which had the CFD (see Figure 2). Figure 6B shows the post-treatment scattergram of the audiometric data for 8 patients who underwent RWR for management of CFD. Six ears had no change in word recognition score (WRS), including the 1 ear with a true conductive hearing loss and CFD (Group 1 Patient 2). One had an improvement of speech discrimination ability from 96 to 100%, while another had a decrease in speech discrimination from 96 to 92%. Including the ear of Group 1 Patient 2 with the conductive hearing loss and CFD, the mean pre-operative air-conduction 4-frequency pure tone average was 19.7 dB (range 5–56.25 dB [SE 7.1]), while the mean post-operative air-conduction 4-frequency pure tone average was 22.8 dB (range 5–51.25 dB [SE 5.2]). As shown in the scatterplot (Figure 6B), 5 ears had worsening of the 4-frequency air-conduction pure tone average; while 3 stayed the same or improved post-operatively. There was no statistically significant difference in the 4-frequency air-conduction pure tone average pretreatment compared to post-treatment (paired *t*-test, *p* = 0.472). Six ears had no change in WRS, including the 1 ear with a true conductive hearing loss and CFD (Group 1 Patient 2). One patient had an improved WRS (96–100%) and one patient had a worsened WRS (96–92%). There was no statistically significant difference in the WRS pretreatment compared to post-treatment (paired *t*-test, *p* = 0.402).

#### Cervical Vestibular Evoked Myogenic Potentials

The cVEMP thresholds are shown in Table 1 for the 8 CFD and 1 CT– TWS patients in Group 1 who had RWR surgery. For all 9 ears receiving RWR, the mean cVEMP threshold was 77.2 dB nHL (SD 7.6, range 70–95 dB nHL), excluding the threshold of 95 dB nHL for the single ear with a CFD and a conductive hearing loss of 42.5 dB pre-operatively, the mean cVEMP threshold was 75 dB nHL (SD 3.8, range 70–80 dB nHL). For the non-operated ears reported in Table 1, the mean cVEMP threshold was 85.7 dB nHL (SD 10.6, range 70–95 dB nHL). Using a Pairwise Comparison, the unoperated ear cVEMP threshold compared to the operated ear (excluding the large conductive hearing loss cVEMP threshold) the mean difference was 10.7 dB nHL (SE 4.0). This was a statistically significant difference (*p* = 0.013). By a Tukey HSD (honestly significant difference) reduced cVEMP threshold was also statistically lower (*p* = 0.034).

The cVEMP thresholds are shown in Table 1 for the 8 CFD patients in Group 2 who did not have RWR surgery. For the 8 ears with CFD in Group 2, the mean cVEMP threshold was 76.9 dB nHL (SE 3.0, SD 8.43; range 70–95 dB nHL). For the 5 ears with no CFD the mean cVEMP threshold was 83.0 dB nHL (SE 3.0, SD 6.71; range 80–95 dB nHL). There was no difference in these thresholds using an independent *t*-test of all values (*p* = 0.199).

As shown in Table 1, the amplitudes of the cVEMP responses, in general declined with age. There was also variability of amplitude in the CFD ear relative to the ear without CFD. There were 2 patients in Group 1 who had post-operative cVEMP studies. In patient 1, the cVEMP response was not present in the operated ear after RWR. In patient 4, in the CFD (right) side the amplitude increased from 358 to 403 μV post-operatively and the threshold remained unchanged at 95 dB pre-operatively and post-operatively. This side had a large conductive hearing loss pre-operatively and post-operatively. For the CT– TWS (left) side the amplitude decreased from 466 to 153 μV post-operatively and the threshold normalized from 75 dB pre-operatively to 95 dB post-operatively.

#### Electrocochleography

As shown in Table 1, only 2 ears in Group 1 had abnormal ECoG data suggestive of ELH (SP/AP ratio >0.42). Both of these subjects underwent RWR procedures. One of these subjects (Table 1, Patient 2) had electrophysiologic evidence of ELH in his CT– TWS left ear (SP/AP ratio 0.43), while his right ear with the CFD (Figure 2) had no evidence of ELH (SP/AP ratio of 0.36). The other subject (Table 1, Patient 3, Figure 1) had electrophysiologic evidence of ELH in her right CFD ear (SP/AP ratio 0.46), while her left ear with the near-CFD (Figure 1) and no TWS symptoms (sound-induced dizziness and headache; autophony [hearing her eyes blinking, chewing sounding loud in her right ear and hearing her heel strike while walking]), had no evidence of endolymphatic hydrops (SP/AP ratio of 0.38).

## Discussion

The present report represents the first description of clinical features (Tables 1, 2) and outcomes of CFD managed surgically with RWR (Figures 3–6) and the largest cohort of patients reported to date with CFD who have not had surgical intervention (Tables 1, 2). In the clinical context of TWS, the latter group have decided that the risk of deafness and facial paralysis for a direct surgical plugging of the CFD third window outweighs the perceived impact of the TWS symptoms on their lives. The RWR approach is an alternative surgical procedure to relieve the TWS symptoms with a low risk of morbidity. Although RWR has the potential to change the biomechanical properties of one of the two natural windows (the round window), we found no statistically different hearing outcomes after RWR in our CFD cohort (Figures 5, 6). Further, the efficacy of the procedure in resolving symptoms was demonstrated by clinically meaningful improvement on the DHI and HIT-6 outcome measures (Figures 3, 4, respectively), as well as captured in the pre- and post-operative patient videos (15–19).

### Advances in Our Understanding of Third Window Syndrome

Over the past 60 years, we have learned much regarding the clinical features, outcomes measured by validated survey instruments and neuropsychology testing as well as objective diagnostic studies in TWS (2–43). Poe's group observed that 94% of patients with SSCD, or symptoms consistent with SSCD, experienced autophony and aural fullness, while 86% were found to have pseudoconductive hearing loss (20, 21). Interestingly, in their 2007 study, they included four cases of CT– TWS among their series of CT+ SSCD who had also had abnormally low cVEMP thresholds (21). Because of their diagnostic dilemma, they did not manage these patients with surgical intervention. The Wackym group has used the Dizziness Handicap Inventory (DHI), the Headache Impact Test (HIT-6) and comprehensive neuropsychology test batteries pre-operatively and post-operatively to measure the cognitive dysfunction and migraine headache in TWS patients to quantify their dysfunction and recovery outcomes (12–19). Crane and coworkers also reported the reduction of DHI scores after plugging the superior semicircular canal in patients with SSCD (40).

In addition, the Wackym group has reported a delayed development of CT– TWS after surgical plugging and resurfacing of CT+ SSCD TWS (12–14). In a series of near-SSCD patients undergoing plugging and resurfacing procedures at the Johns Hopkins Hospital, all patients noted initial improvement in at least one presenting TWS symptom; however, five subjects (45%) reported the persistence or recurrence of at least one TWS symptom at >1 month after surgery (57). In a larger series of SSCD patients, John Carey's group reported that among 222 patients who underwent plugging procedures for SSCD, there were 21 patients who underwent 23 revision surgeries for failure to resolve their TWS symptoms (58). After revision surgery, TWS symptoms were completely resolved in eight (35%), partially resolved in seven (30%), and unresolved in seven (30%) (58). One possible explanation of these findings is that in 14 (61%) of these patients, they also had CT– TWS. It has been suggested that the modiolus may be one site for a CT– TWS (12–14), and Ilmari Pyykkö's and Dennis Poe's demonstration that intratympanic injection of gadolinium subsequently fills the perilymphatic space in mice (59), rats (60), and then exits the inner ear via the modiolus and into the internal auditory canal supports this possibility. Manzari and Scagnelli reported a patient with bilateral SSCD and bilateral dehiscent modioli experiencing bilateral TWS; however, the patient was lost to follow up before surgical intervention (31). Another possible etiology of “CT– TWS” is an unrecognized CFD, as this report underscores.

Naert et al. performed a systematic review of reports of SSCD symptoms and aggregated the most common symptoms into a 22-item common symptom set (41). Among patients with TWS, the same or similar spectrum of symptoms, signs on physical examination and audiological diagnostic findings can be encountered regardless of the site of dehiscence with SSCD, CFD, cochlea-internal carotid artery dehiscence, cochlea-internal auditory canal dehiscence, modiolus, posterior semicircular canal dehiscence, lateral semicircular canal dehiscence, posterior semicircular canal-jugular bulb dehiscence, vestibule-middle ear dehiscence, lateral semicircular canal-facial nerve dehiscence, wide vestibular aqueduct, post-traumatic hypermobile stapes footplate, otosclerosis with internal auditory canal involvement and in patients with CT– TWS. Table 3 summarizes the spectrum of symptoms, signs, exacerbating factors, diagnostic tools and metrics seen, and used, in patients with TWS caused by a dehiscence at any site (2–43, 57, 58, 61). An important point is that TWS is a clinical entity that presents a symptom spectrum rather than a uniformly observed set of symptoms. Thus, the clinical presentation of an individual patient with TWS is not specific to the site of dehiscence; high-resolution temporal bone CT is necessary to establish the site of dehiscence. This observation, in turn, dictates the range of management options.

### Cochlea-Facial Nerve Dehiscence and Other Identified Sites of Dehiscence

Although Jyung and colleagues were the first to identify CFD resulting in TWS in 2014, neither of their two patients were managed surgically (28). As interest in this clinical entity producing TWS has increased, there have been three recent studies focused on the histologic, cadaveric micro-CT and clinical CT prevalence of CFD (32, 44, 45). Fang and coworkers at reported that the histologic prevalence of CFD was 0.59% in 1,020 temporal bone specimens (32). They found that the mean cochlea-facial partition width (CFPW) was 0.23 mm (range 0–0.92 mm, SD 0.15 mm). In particular, 35% of the temporal bones had a CFPW <0.1 mm, which would appear as a CFD on high-resolution temporal bone CT due to current radiographic limitations. They also noted a correlation between a smaller cross-sectional otic capsule area (OCA) with thinner CFPW, which they speculated may represent a developmental (or scaling) factor and may place older, female and Caucasian patients at greater risk of having a CFD (32).

The Rask-Andersen group in Uppsala, Sweden reported a higher prevalence of CFD in microdissected human temporal bones (43). Of the 282 molds analyzed for CFD, 1.4% (4/282) were found to have a CFD. They also measured the CFPW in 48 silicone molds and 49 resin molds. In the silicone molds, the mean CFPW was 0.20 mm while in the resin molds, the mean CFPW was 0.22 mm; remarkably similar to the histologically measured CFPW in the Fang et al. study (23, 28). They also found one instance of SSCD (1.25%) and two near-SSCD occurrences (2.5%) in 80 microdissected temporal bones that underwent micro-CT and 3D rendering (43). Nikolas Blevins' group at Stanford University recently reported a higher prevalence of CFD in 206 high-resolution temporal bone CT scans (406 ears), identifying 5.4% of ears (22/406 ears) and 9.2% of (19/206 patients) as meeting criteria for CFD; but only 1.4% (3/206 patients) had bilateral CFD (45). The mean CFPW was 0.6 ± 0.2 mm (range 0–0.8 mm), reflecting the lower resolution of their imaging technology (45). This latter issue is illustrated in Figure 1 where a right near-CFD was seen, yet the patient did not have TWS on the side of the apparent near-CFD. In the Stanford study, they found 33 ears (26 patients, 7 bilateral) with SSCD; of those three ears (2 patients, 1 bilateral) had SSCD and CFD.

The present study identified a fairly high prevalence of otic capsule dehiscence in high resolution temporal images from 401 subjects with TWS symptoms. However, it should be emphasized that all of our patients had TWS symptoms, whereas the status of TWS symptoms was not reported for the subjects in published prevalence studies (32, 44, 45). We identified 463 temporal bones (57.7% [463/802]) with a single site of dehiscence (SSCD, near-SSCD, CT– TWS, CFD, cochlea-internal auditory canal, wide vestibular aqueduct, lateral semicircular canal, modiolus and posterior semicircular canal, SSCD and superior petrosal sinus, SSCD and subarcuate artery). If the CT– TWS temporal bones were excluded, there was single site temporal bone dehiscence found in 366 (366/402 [91.0%]). Regarding multiple sites of dehiscence, there were 38 instances (38/405 [9.38%]) of two site dehiscence (SSCD and CFD, CFD and cochlea-internal auditory canal, CFD and wide vestibular aqueduct, SSCD and cochlea-internal auditory canal, SSCD and posterior semicircular canal-jugular bulb). There was one instance of three sites (3/405 [0.24%]) of dehiscence (SSCD and posterior semicircular canal and wide vestibular aqueduct). The prevalence of multiple-site findings is important to consider when faced with recurrent or incompletely resolved TWS symptoms after plugging a SSCD (12–14, 57, 58). In two of the Johns Hopkins group's publications (57, 58), 45% of their near-SSCD patients and 9.5% of SSCD patients had persistent or recurrent TWS symptoms after surgery via a middle fossa approach and plugging. In light of our recent observations and the histologic, cadaveric and patient CT scan prevalence of CFD and concurrent SSCD and CFD, careful assessment of the presence of CFD in patients with SSCD should be completed and factored into the surgical planning.

Concurrent second otic capsule dehiscence sites in patients with SSCD have been reported previously (12–14, 31, 32, 44, 45). However, because many patients with radiographic evidence of CFD may not have clinical TWS symptoms, the neurotologist author (PAW), does not recommend, or perform, surgical management with RWR of the possible concurrent CFD at the same time as SSCD plugging. It should be noted that even if a SSCD or near-SSCD was found, only about half the patients (52.7%, 175/332) elected to undergo plugging of their SSCD by one of the authors (PAW) between February 2010 and through February 2019. The important point is that surgical management should never be made based solely on the radiographic findings, but rather a combination of objective audiologic test data, clinical symptoms and the measured impact on the patient's life as measured with validated survey instruments, such as the DHI and HIT-6. For many patients, an understanding the source of their TWS symptoms, lifestyle/activity changes and use of an ear plug on the affected side provide sufficient relief for the patient to elect a conservative, non-surgical management approach. The same is true for the other sites of dehiscence found, particularly for CFD. The fact that only 8 patients who had RWR surgery met the inclusion and exclusion criteria used in this study underscores the need to use a comprehensive approach when selecting appropriate surgical candidates.

### Subjects, Validated Survey Instruments and Surgical Intervention

For the CFD patients who had RWR surgery, the efficacy of the procedure was demonstrated by the DHI and HIT-6 outcomes (Figures 3, 4, respectively) for symptomatic relief, particularly of a set of items indicating perceived handicap. The improvement is also captured observationally in the pre- and post-operative patient videos (15–19).

#### Sound-Induced Symptoms

As summarized in Table 1, each cohort included patients who had sound-induced dizziness (gravitational receptor dysfunction/asymmetry type of vertigo). This was observed in 75% (6/8) of the patients in either cohort. It should also be noted in both groups that extreme sound sensitivity/pain was common in the children, but not in adults. In addition to sound-induced dizziness, both TWS groups included patients who had sound-induced headache, agitation, confusion or a sense of being overwhelmed. For those CFD patients who elected not to have RWR surgery, or this was not recommended to them, these symptoms were subjectively not as bothersome to the patients.

#### Hearing Internal Sounds

As summarized in Table 1, the typical spectrum of the perception of internal sounds, seen in other TWS etiologies, was observed in the cohort of CFD who underwent RWR surgery. These included self-reports of their voice sounding resonant, hearing loud chewing sounds, hearing their heartbeat, hearing their heel strikes and/or hearing their eyes move or blink. Of the 8 subjects in Group 1, 37.5% (3/8) could hear their eyes move or blink, which is typical of SSCD and CT– TWS patients (9, 12–21). For the CFD cohort who did not have RWR surgery (Table 1, Group 2) only 12.5% (1/8) were able to hear their eyes move or blink. This difference is likely due to the small sample size. For those CFD patients who had RWR surgery, these symptoms resolved post-operatively. For those CFD patients who elected not to have RWR surgery, or this was not recommended to them, these symptoms did not bother the patients sufficiently to offset the perceived risks of surgery.

#### Trauma, Third Window Syndrome and Perilymphatic Fistula

Victor Goodhill (62) originally, advanced a theory that labyrinthine window ruptures are a possible cause of sudden deafness associated with exertion or trauma. This interest was stimulated by Stroud and Calcaterra's (63) suggestion that increased perilymphatic pressure had caused a window “rupture” in their patients with a “spontaneous” PLF. Over the years the term PLF developed a negative connotation and as described by Hornibrook (64) the evolving controversy produced polarized groups of “believers” and “non-believers” (64–67).

Interestingly there are international and regional differences in the degree of controversy regarding PLF. In the light of our recognition that there are multiple sites where third windows occur in the otic capsule, it is interesting to note that Kohut's definition of a PLF, from over a quarter century ago, still applies to all currently known sites producing a TWS (68); “*A perilymph fistula may be defined as an abnormal opening between the inner ear and the external surface of the labyrinth capsule….”* Hence, a fistula of the otic capsule (Kohut's definition) can occur in any location that is in communication with perilymph, whether a SSCD, CFD, or any of the well-established sites that can result in a TWS. Patients can have a congenital or acquired TWS. Of those with acquired TWS, there is an unknown but well-recognized percentage of patients who only become symptomatic after a pressure-related event. Therefore, it is more relevant today to consider Goodhill's two proposed mechanisms of explosive and implosive forces. “Explosive” would require an increase in cerebrospinal fluid (CSF) pressure, transmitted from the internal auditory meatus (through the modiolus) or by the cochlear aqueduct. The theory proposed that a force, transmitted through an abnormally patent cochlear aqueduct, could rupture the basilar membrane and/or Reisner membrane into the scala vestibuli, and conceivably injure the utricle, saccule, the semicircular canal system, the round window membrane, or the annular ligament of the stapes. However, these forces could also create a TWS at a site that had not yet become a PLF by delivering an impulse force to that anatomically vulnerable site. Conversely, an “implosive” force would be from a Valsalva maneuver causing sudden air pressure increase through the Eustachian tube, which could elicit a sharp increase in intratympanic pressure and rupture of the round window membrane, annular ligament of the stapes or an anatomically vulnerable site in the otic capsule.

Over a quarter century ago, Black et al. reported that the majority of patients, with what he reported to be middle ear PLF, experienced altered cognitive status (64%) and headache (88%) (39). We have described and quantified similar cognitive changes and headache that recover after surgery for SSCD and CT– TWS (12–14). Video recordings of consenting patients or parents before and after intervention help to further document these obvious alterations in ways that complement standardized neuropsychology testing (15–19). All 8 patients in the CFD with RWR cohort had a pre-TWS history of an explosive or implosive force exposure (Table 2). In contrast, only 2 of the 8 patients (25%) in the CFD without RWR surgery group had a history of explosive or implosive forces before presentation (Table 2). It should be noted that the same type of mechanisms producing TBI from blast injuries, head trauma or possibly impulsive acoustic energy delivered to the inner ear can produce a TWS or TWS-like symptoms resulting in inner ear dysfunction and asymmetric otolithic input (12–14, 69, 70).

#### Cervical Vestibular Evoked Myogenic Potentials

The cVEMP thresholds are shown in Table 1 for the 8 CFD and 1 CT– TWS patients who had RWR surgery. For all 9 ears receiving RWR, the mean cVEMP threshold was 77.2 dB nHL (SD 7.6, range 70–95 dB nHL). Excluding the threshold of 95 dB nHL for the single ear with a CFD and a conductive hearing loss of 42.5 dB pre-operatively, the mean cVEMP threshold was 75 dB nHL (SD 3.8, range 70–80 dB nHL). For the non-operated ears reported in Table 1, the mean cVEMP threshold was 85.71 dB nHL (SD 10.6, range 70–95 dB nHL). Within the operated CFD subjects, the mean difference between the unoperated ear cVEMP threshold compared to the operated ear (excluding the large conductive hearing loss cVEMP threshold) was 10.71 dB nHL (SE 4.0), which was statistically significant (*p* = 0.013, Tukey HSD test). By a Tukey HSD the reduced cVEMP threshold was also statistically lower (Tukey HSD test, *p* = 0.034). In the SSCD literature, the cVEMP threshold has been reported to be reduced in most patients, but the cVEMP response can be absent or without a reduced threshold, despite surgical confirmation of the SSCD (9, 11–14, 21). In Minor's 2005 series of 65 SSCD patients (11), the mean reduced threshold for the cVEMP was 81 ± 9 dB nHL—which means there would likely be an unknown, but certain percentage of his patients with SSCD who would not meet the 70 dB nHL threshold standard that some clinicians have advocated anecdotally and would be categorized as “negative.” Thus, what might appear to be a “discrepancy” is well-described in the SSCD literature and should be factored into the decision-making when managing patients with TWS due to CFD.

The cVEMP thresholds are shown in Table 1 for the 8 CFD patients in Group 2 who did not have RWR surgery. For the 8 ears with CFD the mean cVEMP threshold was 76.9 dB nHL (SE 3.0, SD 8.4; range 70–95 dB nHL). There was 1 ear with a CFD that had no cVEMP response (Table 1). For the 5 ears with no CFD the mean cVEMP threshold was 83.0 dB nHL (SE 3.0, SD 6.71; range 80–95 dB nHL). There was no difference in these thresholds using an independent *t*-test of all values (*p* = 0.199).

As shown in Table 1, the amplitudes of the cVEMP responses, in general declined with age. There was also variability of amplitude in the CFD ear relative to the ear without CFD. Noij et al. found that in SSCD patients, the threshold audiometry and cVEMP data were useful diagnostically and for monitoring outcomes post-operatively, these measures showed no significant correlation with vestibular and most auditory symptoms or their severity (71).

Because air-conduction cVEMP studies were performed and soft tissue and cartilage were placed in the middle ear and over the RW, post-operative cVEMP studies were not routinely performed in the cohort of patients who had CFD and underwent RWR (Group 1). However, there were 2 of these patients who had post-operative cVEMP studies. In patient 1, the cVEMP response was not present in the operated ear after RWR. In patient 4, in the CFD (right) side the amplitude increased from 358 to 403 μV post-operatively and the threshold remained unchanged at 95 dB pre-operatively and post-operatively. This side had a large conductive hearing loss pre-operatively and post-operatively. For the CT– TWS (left) side the amplitude decreased from 466 to 153 μV post-operatively and the threshold normalized from 75 dB pre-operatively to 95 dB post-operatively.

#### Dizziness Handicap Inventory

For the CFD cohort who had RWR procedures performed (Tables 1, 2, Group 1), there was a highly statistically significant (*p* < 0.0001) (Figure 3) improvement in the mean DHI score. For the CFD cohort who did not choose to have surgery (Tables 1, 2, Group 2), statistical comparison of Group 1 (CFD with RWR) to Group 2 (CFD without RWR) revealed that the DHI scores were no different between the groups (*p* > 0.05) (Tables 1, 2).

We tested the hypothesis that there are significant differences in the first symptom report scores (patterns) between the two patient groups to determine if there were any significant differences between symptom item endorsements in patients that may be related to election of surgery. There were 3 questions related to perceived handicap that were significantly larger in the group electing surgery: “*Does performing more ambitious activities, such as sports, dancing, household chores (sweeping or putting dishes away) increase your problems?”* (P8), “*Because of your problem, is it difficult for you to do strenuous homework or yard work?”* (F14) and “*Because of your problem, do you feel handicapped?”* (E21). By binary logistic regression (Wald criterion), responses to DHI items P4 (“*Does walking down the aisle of a supermarket increase your problems?”*) and F14 (“*Because of your problem, is it difficult for you to do strenuous homework or yard work?”*) were sufficient to classify 7 of 8 of each group correctly, with F14 alone producing a correct classification of 6 of 8 from each group. Based on these findings, the decision to have RWR surgery for CFD appears to be a function of the perceived handicap related to the difficulty of performing tasks that require exertion.

### Migraine Headache and Outcomes After Round Window Reinforcement

For the CFD cohort who had RWR procedures performed (Tables 1, 2, Group 1), there was a highly statistically significant (*p* < 0.001) (Figure 4) improvement in the HIT-6 score post-operatively. Statistical comparison of Group 1 (CFD with RWR) to Group 2 (CFD without RWR) revealed that the HIT-6 scores were no different between the groups (*p* > 0.05) (Tables 1, 2).

It is approaching one-half century ago that Gordon (67) hypothesized that the migraine headaches seen in PLF patients are caused by reduced spinal fluid pressure. The series published by Black and colleagues (39) 88% of their PLF patients experienced headache. In our longitudinal study of cognitive dysfunction and recovery in TWS, we found that migraine headaches were present in 88% (7/8) of subjects with CT– TWS only, 100% (4/4) of subjects with SSCD and subsequent CT– TWS, and 80% (4/5) of subjects with SSCD only (13). We also reported migraine variants that can occur with the migraine headaches or as separate episodes, including all three variants; ocular migraine, hemiplegic migraine and VM (12–14). We hypothesize that migraines are triggered by the abnormal otolithic input much in the same way that some migraine patients have migraines triggered by trigeminal stimulation. Removal of the abnormal otolithic input would eliminate a trigger, leading to either resolution or improvement of the migraines to the extent that medical management is then successful. Removing the abnormal otolithic input in CFD is achieved by RWR via returning to a two mobile window state rather than the TWS state.

Vestibular migraine (VM), also termed migraine-associated dizziness, has become recognized as a distinct clinical entity that accounts for a high proportion of patients with vestibular symptoms [for review see Furman et al. (72)]. A temporal overlap between vestibular symptoms, such as vertigo and head-movement intolerance, and migraine symptoms, such as headache, photophobia, and phonophobia, is a requisite diagnostic criterion. Physical examination and laboratory testing are usually normal in VM but can be used to rule out other vestibular disorders with overlapping symptoms, such as with the various defects associated with TWS. Vestibular migraine patients typically do not have sound-induced dizziness and nausea or autophony. However, when these patients have endolymphatic hydrops, they can have sound sensitivity that borders on a Tullio phenomenon. For this reason, when a high-resolution temporal bone CT shows no evidence of a bony dehiscence, all patients suspected as having CT– TWS should be treated as a VM patient, since medical management, if successful, avoids unnecessary surgery (12–14). This management strategy is also used by the neurotologist author (PAW) for patients suspected of having a clinically relevant CFD. It should be noted that all 8 patients who elected to undergo RWR for their TWS secondary to their CFD were all treated as vestibular migraine patients before surgery. The mean duration of medical management was 25.2 months (range 2.5–85 months).

Vestibular migraine is an example of the integral overlap between vestibular pathways and migraine circuit triggers and central mechanisms for premonitory symptom generation. Information transmitted by peripheral vestibular sensory organs and the vestibular nerve to the medulla and pons is an external trigger within the migraine circuit construct proposed by Ho and coworkers (73). This model is based upon the distribution of the neuropeptide calcitonin-gene-related-peptide (CGRP), which has a complex distribution within the vestibular periphery (74). One must acknowledge that the use of CGRP-binding monoclonal antibodies as biologics in the clinical practice of migraine management (75, 76) has a potential to produce a side effect of peripheral vestibular dysfunction or injury due to the impairment of vestibular efferent function.

Migraine headache is nearly always present in patients with gravitational receptor dysfunction type of vertigo caused by TWS. Our study shows that it may also accompany CFD. Migraine seem to be less frequent with rotational receptor dysfunction type of true rotational vertigo (12–19, 39). This is an important concept as CT– TWS, SSCD, and CFD can be associated with three variants of migraine: hemiplegic migraine, ocular migraine and vestibular migraine (12–14, 77). As shown in Table 2, all 8 subjects (100%) had migraine headaches and 5/8 (62.5%) CFD patients undergoing RWR experienced migraine variants before surgery (3 CFD patients had intermittent VM episodes and less frequent ocular migraines, while 2 CFD patients had intermittent ocular migraines). For the CFD patients who did not undergo RWR (Table 2), 7/8 (87.5%) had migraine headaches and 4/8 (50%) of patients with CFD experienced migraine variants before surgery (2 CFD patients had intermittent VM episodes and less frequent ocular migraines, while 2 CFD patients had VM). In patients with CFD and TWS, the VM episodes can produce a combination of infrequent true rotational vertigo attacks on a background of a gravitational receptor (otolithic) dysfunction type of vertigo. The post-operative HIT-6 results document a profound amelioration of reported headache symptoms in these CFD cases after RWR (Figure 4). Because migraine has a high incidence and there are multiple trigger mechanisms, there may only be a marked decrease of the frequency and intensity of the migraines in other cases, but it is often the case that once patients have reached this point they can improve to the point that they come under control with medical management (12–19, 77).

### Cognitive Dysfunction and Recovery

#### Memory, Attention, and Executive Function

Patients with TWS also report symptoms consistent with cognitive dysfunction, spatial disorientation, anxiety and autonomic dysfunction. The degree that these functions and symptoms were impacted in our two cohorts varied as summarized in Table 2. A broader description of the range of symptoms and measurement tools available is summarized in Table 3.

One possible hypothesis of why these TWS patients experience their cognitive dysfunction and spatial disorientation and recovery of function after surgical intervention is that intermittent aberrant otolithic input to the cerebellum creates an episodic but reversible cerebellar cognitive affective syndrome (78–80). Schmahmann conceptualizes cerebellar cognitive affective syndrome as dysmetria of thought and emotion. He describes impairment of executive function (planning, set-shifting, verbal fluency, abstract reasoning and working memory); spatial cognition (visual spatial organization and memory); personality change (blunting of affect or disinhibited and inappropriate behavior); and language deficits (agrammatism and aprosodia) (78–80). These clinical features closely fit what TWS patients describe and their neuropsychology testing measures (12–19). To varying degrees, TWS patients describe cognitive dysfunction (impaired memory and concentration, word finding and name finding difficulty, occasional slurred speech and for women, the loss of the ability to listen to more than one person speaking at time), spatial disorientation (trouble judging distances, sense of detachment, sometimes perceiving the walls moving/breathing or the floor moving, and less commonly out of body experiences), and anxiety (sense of impending doom). In children and young adults continuing their education, their academic performance typically drops; they miss days of school and are often assigned a psychiatric or neurobehavioral diagnosis (12–19). These symptoms are summarized in Table 2 for our two cohorts of CFD patients.

In addition, normal vestibular information appears to be important for head direction responses cells in pathways involving the anterodorsal thalamic nucleus. For example, Yoder and Taube (81) showed that the head direction responses are highly abnormal in a genetic mutant mouse without otolith function. The disruption is expected to extend throughout navigation-related pathways, including the hippocampal formation. We suggest that aberrant vestibular information from a unilateral TWS may also lead to disruption of a variety of cognitive processes by disrupting similar responses in our patients. Similar mechanisms may be involved with degraded otolith function in other contexts, for example in aging (82).

In earlier studies of patients undergoing surgery for CT+ SSCD TWS, CT– TWS as well as patients who had surgery for CT+ SSCD TWS and had surgery for a subsequent CT– TWS, we reported impaired executive function and Wide Range Assessment of Memory and Learning, Second Edition (WRAML2) domain abnormalities in these patients pre-operatively and that there was resolution post-operatively (13). We used the DKEFS and found that there was significant post-operative improvement in both the Delis-Kaplan Executive Function System (DKEFS) Motor Speed score [*F*_(2,28)_ = 10.31, *p* < 0.01] and the Number-Letter Switching score [*F*_(2,28)_ = 6.04, *p* < 0.05] (4, 7). These findings are consistent with our hypothesis that aberrant vestibular input in TWS can contribute to signs and symptoms in the cognitive domain.

The role of migraine in these TWS patients may also contribute to the observed cognitive dysfunction and depression. As reviewed by Ravishankar and Demakis (83), research has shown that migraine can affect verbal, visual memory, and selective attention tasks. Cognitive impairments observed in migraineurs have been found to occur during a migraine attack, after the attack, and even when the individual does not exhibit any residual effects of the attack. Individuals with migraine are at a greater risk of developing anxiety and depression. However, the relatively long delay in recovery of cognitive function after surgery for TWS argues against migraine as the cause of the cognitive dysfunction in these patients (13).

Our comorbidity study (14) noted a high rate of psychological comorbidity (*n* = 6). The Millon Behavioral Medicine Diagnostic (MBMD) and the clinical psychology examinations were the most useful in identifying these comorbidities (14). Factitious disorder, functional neurologic symptom disorder (formerly conversion disorder) dissociative motor disorder variant, somatic symptom disorder, attention deficit hyperactivity disorder (ADHD), dissociative identity disorder (DID), major depressive disorder (MDD), and post-traumatic stress disorder (PTSD) were represented in 6 of the 12 participants in the comorbidity cohort. Suicidal ideation was also common (*n* = 6) (14). These findings underscore the challenges in sorting out the TWS symptoms caused by the dehiscence, those resulting from other comorbid conditions, or those resulting from interactions between the two factors. Clinically, we have incorporated a staged approach to assessing our TWS patients for comorbidities using baseline cognitive screening with the Montréal Cognitive Assessment (MoCA) as well as depression and anxiety scales. Pre-operative patients also undergo a comprehensive neuropsychological evaluation covering the domains of motor speed, complex attention, processing speed, executive functioning, language, visuospatial abilities, memory, and mood/personality. Results are utilized for a thorough diagnostic differential as well as to identify comorbid factors that may complicate post-surgical outcomes, such as personality disorders and chronic psychiatric illness. Post-operative neuropsychological reevaluations (covering the aforementioned domains with alternative forms) occur at 6–8 months after surgery to determine cognitive symptom improvement as well as to identify residual deficits that may be amenable to neurorehabilitation.

### Hearing Outcomes and Electrocochleography

#### Hearing Outcomes

The magnitude of the pseudoconductive hearing loss was, in general smaller than that seen in cases of SSCD. However, our sample size is much smaller than most series of SSCD addressing this question. The pseudoconductive hearing loss is not always present or can be small in SSCD patients (11–13, 21) and in Poe's series of 65 patients published in 2007 (21), 86% had a pseudoconductive hearing loss, while 14% did not. In Minor's 2005 series, only 70% had a pseudoconductive hearing loss of 10 dB or greater while 30% did not (11). In the current series there was no statistically significant difference in the 4-frequency air-bone gap pretreatment compared to post-treatment (*p* = 0.091) (Figure 5B). There was no statistically significant difference in the WRS pretreatment compared to post-treatment (paired *t*-test, *p* = 0.402) (Figure 6). While there was no significant difference in the air-bone gap due to the pseudoconductive loss and the post-operative additional conductive hearing loss due to the RWR and the associated 4-frequency pure tone average air-conduction thresholds, when counseling patients considering RWR for CFD, it is typically the case that their other TWS symptoms are so severe that they would be willing to sacrifice hearing to eliminate or reduce their TWS symptoms. Fortunately, our data suggests that they are unlikely to experience this negative hearing outcome after RWR.

#### Electrocochleography

As shown in Table 1, only 2 ears had abnormal electrocochleography suggestive of ELH (SP/AP ratio >0.42). Both of these subjects underwent RWR procedures. One of these subjects (Table 1, Group 1 Patient 2) had electrophysiologic signs of endolymphatic hydrops in his CT– TWS left ear (SP/AP ratio 0.43), while his right ear with the CFD (Figure 2) had no evidence of endolymphatic hydrops (SP/AP ratio of 0.36). The other subject (Table 1, Group 1 Patient 3) had electrophysiologic signs of ELH in her right CFD ear (SP/AP ratio 0.46), while her right ear with the near-CFD (Figure 1) and no TWS symptoms, had no evidence of ELH (SP/AP ratio of 0.38). This finding is very different than what is observed in patients with SSCD (12, 13, 57). Arts and colleagues at the University of Michigan were the first to report reversible abnormal ECoG/ELH in patients with SSCD (57). Fourteen of 15 ears confirmed to have SSCD on CT imaging were found to have ECoG evidence of ELH. In all 4 patients who underwent plugging of the SSCD, the ECoG SP/AP ratio normalized post-operatively (57).

### Study Limitations

Although this was a retrospective study with a small sample size (*n* = 16), it is much larger than the 2 published cases of CFD in patients experiencing TWS. There are an additional 5 cases of CFD reported in the context of facial nerve stimulation in cochlear implant recipients. In our study, while cognitive dysfunction, spatial disorientation and anxiety were reported by the patients (Table 2), and in many cases captured by their pre-operative videos, objective measurements of these symptoms of TWS were not uniformly or consistently performed, although many underwent formal neuropsychology testing. In addition, tools to measure spatial disorientation and anxiety were not incorporated into their clinical care, so these metrics were not available to compare the patients who underwent RWR surgery and those who did not elect to undergo surgery. Likewise, these metrics were not available to assess outcomes after RWR surgery. The retrospective analysis, though, documents significant clinical features of CFD in patients experiencing TWS that need to be considered in prospective study design.

### Conclusions

Overall there was a marked and clinically significant improvement in DHI, HIT-6, and TWS symptoms post-operatively for the CFD cohort who had RWR surgery. A statistically significant reduction in cVEMP thresholds was observed in patients with radiographic evidence of CFD. Surgical management with RWR in patients with CFD was associated with improved symptoms and outcomes measures. There was no statistically significant change of hearing in the patients with CFD who underwent RWR. It is emphasized that radiographic CFD is not in itself an indication for surgery and that the most important factor in decision-making should be in the context of clinical symptoms and other diagnostic findings. There are three important presenting symptoms and physical findings that are critical when identifying a TWS, including CFD: (1) sound-induced dizziness; (2) hearing internal sounds; and (3) hearing or feeling low frequency tuning forks in an involved ear when applied to a patient's knee or elbow. Another important observation in the study was that multiple sites of dehiscence in temporal bones with TWS occurs and this finding is important to consider when faced with recurrent or incompletely resolved TWS symptoms after plugging a SSCD.

## Data Availability Statement

The datasets generated for this study are available on request to the corresponding author.

## Ethics Statement

The studies involving human participants were reviewed and approved by Rutgers New Brunswick Health Sciences Institutional Review Board (Pro2019000726). Written informed consent from the participants' legal guardian/next of kin was not required to participate in this study in accordance with the national legislation and the institutional requirements. Written informed consent was obtained from the individual(s), and minor(s)' legal guardian/next of kin, for the publication of any potentially identifiable images or data included in this article.

## Author Contributions

PAW and CDB contributed the conception and design of the study. PAW organized the database. CDB performed the statistical analysis. PAW and CDB wrote the first draft of the manuscript. PAW, CDB, PZ, DAS, and JSH wrote the sections of the manuscript. All authors contributed to the manuscript revision, read, and approved the submitted version.

### Conflict of Interest

The authors declare that the research was conducted in the absence of any commercial or financial relationships that could be construed as a potential conflict of interest.

## References

[1] Tullio P Das Ohr und die Entstehung der Sprache und Schrift. Berlin: Urban & Schwarzenberg (1929). p. 1–455.

[2] Huizinga E. The physiological and clinical importance of experimental work on the pigeon's labyrinth. J Laryngol Otol. (1955) 69:260–8. 10.1017/S0022215100050635 14368097

[3] Grieser BJ Kleiser L Obrist D. Identifying mechanisms behind the Tullio phenomenon: a computational study based on first principles. J Assoc Res Otolaryngol. (2016) 17:103–18. 10.1007/s10162-016-0553-0 26883248 PMC4791416

[4] Fox EJ Balkany TJ Arenberg IK. The Tullio phenomenon and perilymph fistula. Otolaryngol Head Neck Surg. (1988) 98:88–9. 10.1177/019459988809800115 3124057

[5] Pyykkö I Ishizaki H Aalto H Starck J. Relevance of the Tullio phenomenon in assessing perilymphatic leak in vertiginous patients. Am J Otol. (1992) 13:339–42. 1415497

[6] Colebatch JG Rothwell JC Bronstein A Ludman H. Click-evoked vestibular activation in the Tullio phenomenon. J Neurol Neurosurg Psychiatry. (1994) 57:1538–40. 10.1136/jnnp.57.12.1538 7798988 PMC1073240

[7] Ostrowski VB Hain TC Wiet RJ. Pressure-induced ocular torsion. Arch Otolaryngol Head Neck Surg. (1997) 123:646–9. 10.1001/archotol.1997.01900060098017 9193230

[8] Weinreich HM Carey JP. Perilymphatic fistulas and superior semi-circular canal dehiscence syndrome. Adv Otorhinolaryngol. (2019) 82:93–100. 10.1159/000490276 30947173

[9] Ward BK Carey JP Minor LB. Superior canal dehiscence syndrome: lessons from the first 20 years. Front Neurol. (2017) 8:177. 10.3389/fneur.2017.00177 28503164 PMC5408023

[10] Minor LB Solomon D Zinreich JS Zee DS Sound- and/or pressure-induced vertigo due to bone dehiscence of the superior semicircular canal. Arch Otolaryngol Head Neck Surg. (1998) 124:249–58. 10.1001/archotol.124.3.249 9525507

[11] Minor LB. Clinical manifestations of superior semicircular canal dehiscence. Laryngoscope. (2005) 115:1717–27. 10.1097/01.mlg.0000178324.55729.b7 16222184

[12] Wackym PA Wood SJ Siker DA Carter DM. Otic capsule dehiscence syndrome: superior canal dehiscence syndrome with no radiographically visible dehiscence. Ear Nose Throat J. (2015) 94:E8–24. 10.1177/014556131509400802 26322461

[13] Wackym PA Balaban CD Mackay HT Wood SJ Lundell CJ Carter DM. Longitudinal cognitive and neurobehavioral functional outcomes after repairing otic capsule dehiscence. Otol Neurotol. (2016) 37:70–82. 10.1097/MAO.0000000000000928 26649608 PMC4674143

[14] Wackym PA Mackay-Promitas HT Demirel S Gianoli GJ Gizzi MS Carter DM. Comorbidities confounding the outcomes of surgery for third window syndrome: outlier analysis. Laryngoscope Invest Otolaryngol. (2017) 2:225–53. 10.1002/lio2.89 29094067 PMC5654938

[15] Wackym PA Round Window Reinforcement Surgery for Cochlea-Facial Nerve Dehiscence: Symptoms and Testing. Available online at: https://youtu.be/2z1RJEKZQ1A (accessed November 11, 2019).

[16] Wackym PA Cochlea-Facial Nerve Dehiscence: Traumatic Third Window Syndrome After a Snowboarding Accident. Available online at: https://youtu.be/NCDMD5FGf-w (accessed November 11, 2019).

[17] Wackym PA Right Cochlea-Facial Nerve Dehiscence: 16 Year Old Thought to Have Conversion Disorder. Available online at: https://youtu.be/fTjsnnUALBw (accessed November 11, 2019).

[18] Wackym PA Surgery for Cochlea-Facial Nerve Dehiscence: Symptoms and Tuning Fork Testing. Available online at: https://youtu.be/lFR-zdYlIsY (accessed November 11, 2019).

[19] Wackym PA Cochlea-Facial Nerve Dehiscence: Third Window Syndrome After a Car Accident. Available online at: https://youtu.be/eJX2RA3okKc (accessed November 11, 2019).

[20] Mikulec AA Poe DS McKenna MJ. Operative management of superior semicircular canal dehiscence. Laryngoscope. (2005) 115:501–7. 10.1097/01.mlg.0000157844.48036.e7 15744166

[21] Zhou G Gopen Q Poe DS Clinical and diagnostic characterization of canal dehiscence syndrome: a great otologic mimicker. Otol Neurotol. (2007) 28:920–6. 10.1097/MAO.0b013e31814b25f2 17955609

[22] Young L Isaacson B. Cochlear and petrous carotid canal erosion secondary to cholesteatoma. Otol Neurotol. (2010) 31:697–8. 10.1097/MAO.0b013e31819bd803 19300300

[23] Meiklejohn DA Corrales CE Boldt BM Sharon JD Yeom KW Carey JP Pediatric semicircular canal dehiscence: radiographic and histologic prevalence, with clinical correlations. Otol Neurotol. (2015) 36:1383–9. 10.1097/MAO.0000000000000811 26164444

[24] Park JJ Shen A Loberg C Westhofen M. The relationship between jugular bulb position and jugular bulb related inner ear dehiscence: a retrospective analysis. Am J Otolaryngol. (2015) 36:347–51. 10.1016/j.amjoto.2014.12.006 25701459

[25] Gopen Q Zhou G Poe D Kenna M Jones D. Posterior semicircular canal dehiscence: first reported case series. Otol Neurotol. (2010) 31:339–44. 10.1097/MAO.0b013e3181be65a4 19841602

[26] Bear ZW McEvoy TP Mikulec AA. Quantification of hearing loss in patients with posterior semicircular canal dehiscence. Acta Otolaryngol. (2015) 135:974–7. 10.3109/00016489.2015.1060630 26107020

[27] Elmali M Poltat AV Kucuk H Atmaca S Aksoy A. Semicircular canal dehiscence: frequency and distribution on temporal bone CT and its relationship with the clinical outcomes. Eur J Radiol. (2013) 82:e606–9. 10.1016/j.ejrad.2013.06.022 23906440

[28] Blake DM Tomovic S Vazquez A Lee HJ Jyung RW Cochlear-facial dehiscence–a newly described entity. Laryngoscope. (2014) 124:283–9. 10.1002/lary.24223 23712934

[29] Fujita T Kobayashi T Saito K Seo T Ikezono T Doi K. Vestibule-middle ear dehiscence tested with perilymph-specific protein cochlin-tomoprotein (CTP) detection test. Front Neurol. (2019) 10:47. 10.3389/fneur.2019.00047 30761077 PMC6363674

[30] Manzari L. Multiple dehiscences of bony labyrinthine capsule. A rare case report and review of the literature. Acta Otorhinolaryngol Ital. (2010) 30:317–20. 21808455 PMC3146321

[31] Manzari L Scagnelli P. Large bilateral internal auditory meatus associated with bilateral superior semicircular canal dehiscence. Ear Nose Throat J. (2013) 92:25–33. 10.1177/014556131309200109 23354888

[32] Fang CH Chung SY Blake DM Vazquez A Li C Carey JP. Prevalence of cochlear-facial dehiscence in a study of 1,020 temporal bone specimens. Otol Neurotol. (2016) 37:967–72. 10.1097/MAO.0000000000001057 27203843

[33] Ho ML Moonis G Halpin CF Curtin HD. Spectrum of third window abnormalities: semicircular canal dehiscence and beyond. Am J Neuroradiol. (2017) 38:2–9. 10.3174/ajnr.A4922 27561833 PMC7963676

[34] Koo JW Hong SK Kim DK Kim JS. Superior semicircular canal dehiscence syndrome by the superior petrosal sinus. J Neurol Neurosurg Psychiatry. (2010) 81:465–7. 10.1136/jnnp.2008.155564 20176603

[35] Ionescu EC Al Tamami N Neagu A Ltaief-Boudrigua A Gallego S Hermann R. Superior semicircular canal ampullae dehiscence as part of the spectrum of the third window abnormalities: a case study. Front Neurol. (2017) 8:683. 10.3389/fneur.2017.00683 29312118 PMC5742101

[36] Dasgupta S Ratnayake SAB. Functional and objective audiovestibular evaluation of children with apparent semicircular canal dehiscence–a case series in a pediatric vestibular center. Front Neurol. (2019) 10:306. 10.3389/fneur.2019.00306 31001191 PMC6454049

[37] Merchant SN Rosowski JJ. Conductive hearing loss caused by third-window lesions of the inner ear. Otol Neurotol. (2008) 29:282–9. 10.1097/MAO.0b013e318161ab24 18223508 PMC2577191

[38] Hornibrook J The Balance Abnormality of Chronic Perilymph Fistula. Available online at: https://www.youtube.com/watch?v=2DXgQMnlgbw (accessed November 11, 2019).

[39] Black FO Pesznecker S Norton T Fowler L Lilly DJ Shupert C. Surgical management of perilymphatic fistulas: a Portland experience. Am J Otol. (1992) 13:254–62. 1609855

[40] Crane BT Minor LB Carey JP. Superior canal dehiscence plugging reduces dizziness handicap. Laryngoscope. (2008) 118:1809–13. 10.1097/MLG.0b013e31817f18fa 18622314

[41] Naert L Van de Berg R Van de Heyning P Bisdorff A Sharon JD Ward BK. Aggregating the symptoms of superior semicircular canal dehiscence syndrome. Laryngoscope. (2018) 128:1932–8. 10.1002/lary.27062 29280497

[42] Shim YJ Bae YJ An GS Lee K Kim Y Lee SY. Involvement of the internal auditory canal in subjects with cochlear otosclerosis: a less acknowledged third window that affects surgical outcome. Otol Neurotol. (2019) 40:e186–90. 10.1097/MAO.0000000000002144 30741893

[43] Bae YJ Shim YJ Choi BS Kim JH Koo JW Song JJ “Third window” and “single window” effects impede surgical success: analysis of retrofenestral otosclerosis involving the internal auditory canal or round window. J Clin Med. (2019) 8:E1182 10.3390/jcm8081182 31394873 PMC6723488

[44] Schart-Morén N Larsson S Rask-Andersen H Li H. Anatomical characteristics of facial nerve and cochlea interaction. Audiol Neurootol. (2017) 22:41–9. 10.1159/000475876 28628917

[45] Song Y Alyono JC Bartholomew RA Vaisbuch Y Corrales CE Blevins NH. Prevalence of radiographic cochlear-facial nerve dehiscence. Otol Neurotol. (2018) 39:1319–25. 10.1097/MAO.0000000000002015 30289844

[46] Fang CH Chung SY Mady LJ Raia N Lee HJ Mary Ying YL Facial nerve stimulation outcomes after cochlear implantation with cochlear-facial dehiscence. Otolaryngol Case Reports. (2017) 3:12–4. 10.1016/j.xocr.2017.04.003

[47] Schart-Morén N Hallin K Agrawal SK Ladak HM Eriksson PO Li H. Peri-operative electrically evoked auditory brainstem response assessment of facial nerve/cochlea interaction at cochlear implantation. Cochlear Implants Int. (2018) 19:324–9. 10.1080/14670100.2018.1481179 29877144

[48] Jacobson GP Newman CW. The development of the Dizziness Handicap Inventory. Arch Otolaryngol Head Neck Surg. (1990) 116:424–7. 10.1001/archotol.1990.01870040046011 2317323

[49] Whitney SL Wrisley DM Brown KE Furman JM. Is perception of handicap related to functional performance in persons with vestibular dysfunction? Otol Neurotol. (2004) 25:139–43. 10.1097/00129492-200403000-00010 15021773

[50] Yang M Rendas-Baum R Varon SF Kosinski M. Validation of the Headache Impact Test (HIT-6™) across episodic and chronic migraine. Cephalalgia. (2011) 31:357–67. 10.1177/0333102410379890 20819842 PMC3057423

[51] Bayliss M Batenhorst A The HIT-6^TM^: A User's Guide. Lincoln, RI: QualityMetric, Inc (2002).

[52] Headache Classification Committee of the International Headache Society (IHS) The international classification of headache disorders, 3rd edition. Cephalalgia. (2018) 38:1–211. 10.1177/0333102417738202 29368949

[53] Gurgel RK Jackler RK Dobie RA Popelka GR. A new standardized format for reporting hearing outcome in clinical trials. Otolaryngol Head Neck Surg. (2012) 147:803–7. 10.1177/0194599812458401 22931898

[54] Wackym PA Tuning Fork Testing in Otic Capsule Dehiscence Syndrome. Available online at: https://www.youtube.com/watch?v=Szp_kO8oVos (accessed November 11, 2019).

[55] Wackym PA Ratigan JA Birck JD Johnson SH Doornink J Bottlang M. Rapid cVEMP and oVEMP responses elicited by a novel head striker and recording device. Otol Neurotol. (2012) 33:1392–400. 10.1097/MAO.0b013e318268d234 22935811

[56] Margolis RH Rieks D Fournier EM Levine SE. Tympanic electrocochleography for diagnosis of Ménière's disease. Arch Otolaryngol Head Neck Surg. (1995) 121:44–55. 10.1001/archotol.1995.01890010032007 7803022

[57] Ward BK Wenzel A Ritzl EK Gutierrez-Hernandez S Della Santina CC Minor LB. Near-dehiscence: clinical findings in patients with thin bone over the superior semicircular canal. Otol Neurotol. (2013) 34:1421–8. 10.1097/MAO.0b013e318287efe6 23644303 PMC3740012

[58] Sharon JD Pross SE Ward BK Carey JP. Revision surgery for superior canal dehiscence syndrome. Otol Neurotol. (2016) 37:1096–103. 10.1097/MAO.0000000000001113 27348392

[59] Zou J Zhang W Poe D Zhang Y Ramadan UA Pyykkö I. Differential passage of gadolinium through the mouse inner ear barriers evaluated with 4.7T MRI. Hear Res. (2010) 259:36–43. 10.1016/j.heares.2009.09.015 19818391

[60] Zou J Poe D Ramadan UA Pyykkö I. Oval window transport of Gd-dOTA from rat middle ear to vestibulum and scala vestibuli visualized by *in vivo* magnetic resonance imaging. Ann Otol Rhinol Laryngol. (2012) 121:119–28. 10.1177/000348941212100209 22397222

[61] Arts HA Adams ME Telian SA El-Kashlan H Kileny PR. Reversible electrocochleographic abnormalities in superior canal dehiscence. Otol Neurotol. (2009) 30:79–86. 10.1097/MAO.0b013e31818d1b51 19092559

[62] Goodhill V. Sudden deafness and round window rupture. Laryngoscope. (1971) 81:1462–74. 10.1288/00005537-197109000-00010 5098107

[63] Stroud MH Calcaterra TC. Spontaneous perilymph fistulas. Laryngoscope. (1970) 80:479–87. 10.1288/00005537-197003000-00012 5436969

[64] Hornibrook J. Perilymph fistula: fifty years of controversy. ISRN Otolaryngol. (2012) 2012:281248. 10.5402/2012/281248 23724269 PMC3658483

[65] Friedland DR Wackym PA. A critical appraisal of spontaneous perilymphatic fistulas of the inner ear. Am J Otol. (1999) 20:261–76. 10100535

[66] Deveze A Matsuda H Elziere M Ikezono T Diagnosis and treatment of perilymph fistula. In: Lloyd SKW Donnelly NP, editors. Advances in Hearing Rehabilitation. Adv Otorhinolaryngol. (2018) 81:133–45. 10.1159/000485579 29794455

[67] Gordon AG. Perilymph fistula: a cause of auditory, vestibular, neurological and psychiatric disorder. Med Hypotheses. (1976) 2:125–34. 10.1016/0306-9877(76)90067-0 785162

[68] Kohut RI. Perilymph fistulas. Clinical criteria. Arch Otolaryngol Head Neck Surg. (1992) 118:687–92. 10.1001/archotol.1992.01880070017003 1627286

[69] Gianoli GJ Soileau JS Wackym PA. Neurological symptoms in US government personnel in Cuba. JAMA. (2018) 320:603–4. 10.1001/jama.2018.8713 30120472

[70] Michael E Hoffer ME, Levin BE, Snapp H, Buskirk J, Balaban CD. Acute findings in an acquired neurosensory dysfunction. Laryngoscope Invest Otolaryngol. (2019) 4:124–31. 10.1002/lio2.231 PMC6383299 30828629

[71] Noij KS Wong K Duarte MJ Masud S Dewyer NA Herrmann BS. Audiometric and cVEMP thresholds show little correlation with symptoms in superior semicircular canal dehiscence syndrome. Otol Neurotol. (2018) 39:1153–62. 10.1097/MAO.0000000000001910 30124614

[72] Furman JM Marcus DA Balaban CD. Vestibular migraine: clinical aspects and pathophysiology. Lancet Neurol. (2013) 12:706–15. 10.1016/S1474-4422(13)70107-8 23769597

[73] Ho TW Edvinsson L Goadsby PJ. CGRP and its receptors provide new insights into migraine pathophysiology. Nat Rev Neurol. (2010) 6:573–82. 10.1038/nrneurol.2010.127 20820195

[74] Wackym PA Ultrastructural organization of calcitonin gene-related peptide immunoreactive efferent axons and terminals in the rat vestibular periphery. Am J Otol. (1993) 14:41–50. 8424475

[75] Xu D Chen D Zhu LN Tan G Wang HJ Zhang Y. Safety and tolerability of calcitonin-gene-related peptide binding monoclonal antibodies for the prevention of episodic migraine–a meta-analysis of randomized controlled trials. Cephalalgia. (2019) 39:1164–79. 10.1177/0333102419829007 30789292

[76] American Headache Society The American Headache Society position statement on integrating new migraine treatments into clinical practice. Headache. (2019) 59:1–18. 10.1111/head.13456 30536394

[77] Wackym PA Balaban CD Mackay HT Carter DM Migraine headache and the migraine variants of hemiplegic migraine, ocular migraine and vestibular migraine in otic capsule dehiscence syndrome: outcomes after targeted repair. In: Barbara M Filipo R, editors. 7th International Symposium on Menière's Disease and Inner Ear Disorders. Amsterdam: Kugler Publications (2016). p. 77–86.

[78] Schmahmann JD. Disorders of the cerebellum: ataxia, dysmetria of thought, and the cerebellar cognitive affective syndrome. J Neuropsychiatry Clin Neurosci. (2004) 16:367–78. 10.1176/jnp.16.3.367 15377747

[79] Hoche F Guell X Vangel MG Sherman JC Schmahmann JD. The cerebellar cognitive affective/Schmahmann syndrome scale. Brain. (2018) 141:248–70. 10.1093/brain/awx317 29206893 PMC5837248

[80] Schmahmann JD. The cerebellum and cognition. Neurosci Lett. (2019) 688:62–75. 10.1016/j.neulet.2018.07.005 29997061

[81] Yoder RM Taube JS. The vestibular contribution to the head direction signal and navigation. Front Integr Neurosci. (2014) 8:32. 10.3389/fnint.2014.00032 24795578 PMC4001061

[82] Kamil RJ Jacob A Ratnanather JT Resnick SM Agrawal Y. Vestibular function and hippocampal volume in the Baltimore Longitudinal Study of Aging (BLSA). Otol Neurotol. (2018) 39:765–71. 10.1097/MAO.0000000000001838 29889787 PMC5999049

[83] Ravishankar N Demakis GJ. The neuropsychology of migraine. Dis Mon. (2007) 53:156–61. 10.1016/j.disamonth.2007.04.006 17544646

