# Supplementary material for: Third Window Syndrome: Surgical Management of Cochlea-Facial Nerve Dehiscence
Source: Front Neurol. 2019 Dec 13;10:1281. doi: 10.3389/fneur.2019.01281 (PMC6923767; doi:10.3389/fneur.2019.01281)
Supplement: Supplementary file 1 [file Data_Sheet_1.pdf]

Round Window Reinforcement with the Perichondrial and Cartilage Graft Technique. For CFD patients who had RWR and the 1 patient with right-sided CT- TWS (Group 1, Tables 1 and 2), the perichondrial and cartilage graft technique described previously for RWR was ultimately performed in all 8 subjects. The bone was drilled off of the RW niche bony-overhang using a 0.8 mm diamond bur. A Lumenis Spectra II (Lumenis Inc., San Jose, CA) laser was used with a Lumenis® Acculite™ EndoOto™ hand held laser probe (Horn, 24 gauge 20° angled, SubMiniature Type A [SMA] 906 connector, 200 µm). The Selecta II has a red 635nm (<5 mW) He NE aiming beam; and a Q-switched frequency doubled 1064 nm Nd:YAG, (532 nm [green wavelength]) diode-pumped solid state laser as its treatment beam. The specific treatment settings used were: power 1000 mW; pulse duration of 0.3 seconds; and pulse interval of 0.3 seconds. Alternatively, a Lumenis AcuPulse DUO CO_2 laser (10.6 µm wavelength) was used with a handheld single-use FiberLase fiber (500 µm spot size) was used. The specific treatment settings used were: power 1.0 W; SuperPulse; and pulse interval of 0.3 seconds. Either laser was used to denude all of the mucosa around the RW niche and also around the anterior portion of bone surrounding the OW annular ligament. The perichondrium graft was thinned using a fascia press and was placed directly on the surface of the RW membrane and extended onto the denuded otic capsule. A 2-mm conchal cartilage graft was harvested using a 2-mm biopsy punch (Miltex, Inc., York, PA) and then split in half using a 6900 Beaver mini-blade (Beaver-Visitec International, Inc., Waltham, MA) and placed into the perichondrial graft overlying the RW. Loose areolar tissue was minced into 0.25 mm pieces separated into two petri dishes. TISEEL, a two-component fibrin sealant, (Baxter Healthcare Corporation, Westlake Village, CA) was used for coating the pieces. One component was a sealer protein solution that contained human fibrinogen and a synthetic fibrinolysis inhibitor, aprotinin, which helps prevent premature degradation of the fibrin clot. The other component was a human thrombin solution and calcium chloride. Each of these solutions was prepared and kept isolated into petri dishes into which the minced tissue was divided. The latter was then circumferentially placed in a manner of a gasket around the cartilage and onto the perichondrium. Too much tissue was intentionally placed in the RW niche and also adjacent to the stapes knowing that some would be resorbed during the healing and connective tissue remodeling phases. Following reinforcement, the middle ear was filled with Gelfoam and tympanomeatal flap returned to the anatomic position. Strips of dry Gelfoam were placed across the intact skin and the skin of the tympanomeatal flap and a small amount of antibiotic ointment was placed over this. Ofloxacin 0.3% otic solution was then placed into the external auditory canal. No additional dressing materials were required.
